# Harnessing stimuli‐responsive biomaterials for advanced biomedical applications

**DOI:** 10.1002/EXP.20230133

**Published:** 2024-05-30

**Authors:** Ziming Liao, Tingting Liu, Zhimin Yao, Tian Hu, Xiaoyuan Ji, Bin Yao

**Affiliations:** ^1^ Academy of Medical Engineering and Translational Medicine Tianjin University Tianjin P. R. China; ^2^ Division of Engineering in Medicine Department of Medicine Brigham and Women's Hospital Harvard Medical School Cambridge Massachusetts USA; ^3^ Research Center for Nano‐Biomaterials and Regenerative Medicine Department of Biomedical Engineering College of Biomedical Engineering Taiyuan University of Technology Taiyuan Shanxi P. R. China; ^4^ Department of Laboratory Diagnosis The 971th Hospital Qingdao P. R. China; ^5^ Research Center for Tissue Repair and Regeneration Affiliated to the Medical Innovation Research Department PLA General Hospital and PLA Medical College Beijing P. R. China; ^6^ Sichuan Preschool Educators' College Mianyang P. R. China; ^7^ MRC Human Immunology Unit MRC Weatherall Institute of Molecular Medicine, University of Oxford John Radcliffe Hospital Oxford UK

**Keywords:** biomaterials, biomedical, physical stimulation, stimulus‐responsive

## Abstract

Cell behavior is intricately intertwined with the in vivo microenvironment and endogenous pathways. The ability to guide cellular behavior toward specific goals can be achieved by external stimuli, notably electricity, light, ultrasound, and magnetism, simultaneously harnessed through biomaterial‐mediated responses. These external triggers become focal points within the body due to interactions with biomaterials, facilitating a range of cellular pathways: electrical signal transmission, biochemical cues, drug release, cell loading, and modulation of mechanical stress. Stimulus‐responsive biomaterials hold immense potential in biomedical research, establishing themselves as a pivotal focal point in interdisciplinary pursuits. This comprehensive review systematically elucidates prevalent physical stimuli and their corresponding biomaterial response mechanisms. Moreover, it delves deeply into the application of biomaterials within the domain of biomedicine. A balanced assessment of distinct physical stimulation techniques is provided, along with a discussion of their merits and limitations. The review aims to shed light on the future trajectory of physical stimulus‐responsive biomaterials in disease treatment and outline their application prospects and potential for future development. This review is poised to spark novel concepts for advancing intelligent, stimulus‐responsive biomaterials.

## Graphical abstract

The application of physical stimulation biomaterials based on ultrasound, light, electricity, and magnetism in the field of biomedicine is summarized, as electrical stimulation of nerve regeneration, electrical stimulation of skin regeneration, electrical stimulation of bone regeneration, photothermal/photodynamic, ultrasound stimulated tissue repair, ultrasonic controlled release, targeting/controlled release, magnetic stimulation tissue repair.

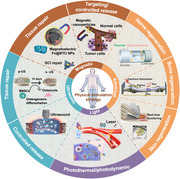

## 1 INTRODUCTION

With the continuous progress of science and technology, people pay more and more attention to medical health and promote the application of more biomaterials in the field of biomedicine.^[^ ^1^ ^]^ It is becoming increasingly clear that these biomaterial systems are not just limited to the passive support of cells.^[^ ^2^ ^]^ Through ingenious design and advanced manufacturing methods, we can introduce the properties of different biomaterials to manipulate cells across time and space, thus achieving better control and application in biomedical engineering. Many endogenous mechanisms play a vital role in regulating cell behavior and the external microenvironment.^[^ ^3^ ^]^ Such as electrophysiological activities, magnetic signals, growth factors, and signal molecules. These factors regulate damage repair, immune response, and cellular behavior.^[^ ^4^, ^5^ ^]^ Therefore, biomaterials with intelligent responses can generate these signals through controlled exogenous stimuli, enabling simpler and more appropriate methods to manipulate cell behavior. Stimuli‐responsive biomaterials can respond to a wide range of external signals, including light, electrical, ultrasonic, and magnetic stimuli, and so on. However, the ideal stimuli‐responsive biomaterials can convert external energy sources into meaningful cellular signals with minimal design complexity.^[^ ^6^ ^]^ One advantage of utilizing this technique is that the stimulation can be precisely regulated through specialized instruments, allowing for accurate control of cell responses as needed.

Moreover, by modifying the size of the external signal, the degree to which the biomaterial responds can be customized, granting greater control than a simple on/off switch. While photoresponsive biomaterials have garnered significant attention in the tissue engineering field, other types of stimuli‐responsive biomaterials remain unexplored in terms of their potential to influence cell fate.^[^ ^7^, ^8^ ^]^ It has been noted that while electrical stimulation is useful for nerve and muscle stimulation, it may not be as effective in promoting pluripotent cell differentiation.^[^ ^9^ ^]^ Ultrasonic stimulation has unique advantages due to its remarkable tissue penetration and remote drive capabilities, which are often used to control drug delivery in vivo, especially for penetrating the blood‐brain barrier. It shows potential as a multifunctional stimulation for remote material operation.^[^ ^10^ ^]^ Magnetically responsive biomaterials are another interesting system that has played a huge role in nanomedicine and has great prospects for achieving multi‐functional collaborative precision medicine.^[^ ^11^ ^]^

**FIGURE 1:**
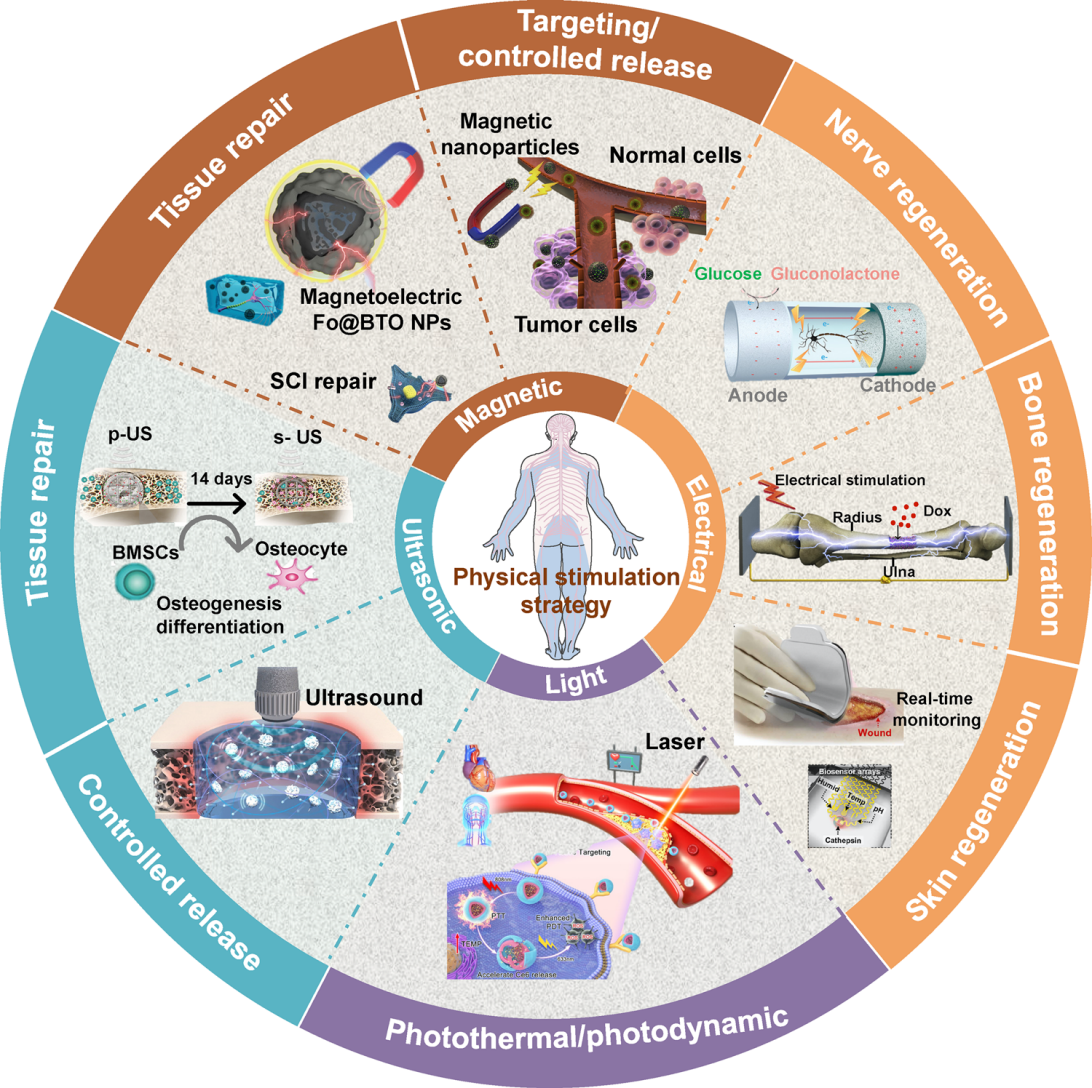
The application of physical stimulation biomaterials based on ultrasound, light, electricity, and magnetism in the field of biomedicine is summarized, as electrical stimulation of nerve regeneration. Reproduced with permission.^[^ ^12^ ^]^ Copyright 2019, John Wiley & Sons. Electrical stimulation of skin regeneration. Reproduced with permission.^[^ ^13^ ^]^ Copyright 2022, Elsevier. Electrical stimulation of bone regeneration. Reproduced with permission.^[^ ^14^ ^]^ Copyright 2020, Elsevier. Photothermal/photodynamic. Reproduced with permission.^[^ ^15^ ^]^ Copyright 2021, American Chemical Society. Ultrasound stimulated tissue repair. Reproduced with permission.^[^ ^16^ ^]^ Copyright 2023, Elsevier. Ultrasonic controlled release. Reproduced with permission.^[^ ^17^ ^]^ Copyright 2023, Elsevier. Targeting/controlled release. Reproduced with permission.^[^ ^18^ ^]^ Copyright 2021, Elsevier. Magnetic stimulation tissue repair. Reproduced with permission.^[^ ^19^ ^]^ Copyright 2021, John Wiley & Sons.

In this review, we conducted a thorough review of physical stimulation‐related biomaterials used in biomedical engineering, as presented in Figure 1. First, we describe the various types and modes of action of physical stimuli. Next, we briefly analyze the principle of the different kinds of stimuli‐responsive biomaterials. Then, we divide the applications of physical stimuli‐responsive biomaterials into four categories: electrical stimulation, light stimulation, ultrasonic stimulation, and magnetic stimulation. Finally, a summary of the physical stimuli‐responsive biomaterials is given, and according to the existing characteristics and existing problems, it is hoped that by identifying the most promising biomaterials and external stimulation combinations for different tissue types in the future, an accurate and controllable tissue engineering platform can be designed and utilized to promote the progress of biomedicine.

## 2 COMMON TYPES OF PHYSICAL STIMULATION

At present, the common physical stimulation in biomedical engineering is mainly divided into electrical stimulation, light stimulation, ultrasonic stimulation, and magnetic stimulation. The most appropriate stimulation method should be selected for different application fields.

### 2.1 Electrical stimulation

Electrical stimulation has several attractive advantages, including non‐invasiveness, high spatiotemporal controllability, and rapid reversible induction.^[^ ^20^ ^]^ Electrical stimulation is mainly divided into bioelectrical stimulation, percutaneous electrical stimulation, implantation electrical stimulation, and radio stimulation.

#### 2.1.1 Bioelectrical stimulation

Bioelectrical stimulation mainly refers to the use of endogenous biological current for electrical stimulation, but the electrical stimulation of this method is weak and uncontrollable, and the stimulation effect is relatively limited.^[^ ^21^ ^]^

#### 2.1.2 Percutaneous electrical stimulation

Percutaneous electrical stimulation systems include in vitro programmable power generators (typically providing adjustable low‐frequency pulse currents), percutaneous output wires, percutaneous electrodes, percutaneous electrical stimulation systems, percutaneous radiofrequency ablation systems, external radiofrequency ablation systems, and percutaneous radiofrequency therapy systems.^[^ ^22^, ^23^ ^]^ Usually, this method requires a percutaneous wire to be connected to an external bulky electrical stimulation device, but it comes with the risk of infection and a treatment schedule that is not conducive to daily activities. At the same time, specialized medical facilities and trained operators are required to perform daily electrical stimulation therapy, which is considered to take up limited medical resources.^[^ ^24^ ^]^

#### 2.1.3 Implanted electrical stimulation

The implanted electrical stimulation is mainly divided into: 1) internal battery‐powered electrical stimulation; 2) internal bio‐fuel electrical stimulation; and 3) internal physically generated electricity stimulation.

(1) Batteries, as a stable power source, are often used to construct implantable electrical stimulation systems for nerve regeneration. But battery wear and lead leakage can lead to second surgeries, increasing the financial burden on patients and being serious or even life‐threatening.
(2) Biofuel electrical stimulation mainly uses enzyme biological reactions to carry out bioelectrochemical reactions in vivo, converting biomass energy into electric energy used in electrical stimulation.^[^ ^25^, ^26^ ^]^ The stimulation current is formed in the chemical reaction of the Yin and Yang poles in the body, avoiding cumbersome external equipment like percutaneous electrical stimulation and the infection problem that may exist during each electrical stimulation. However, due to its inability to effectively control the reaction occurrence and low electrical stimulation output flow, its practical application is limited.
(3) The application of piezoelectric materials in the construction of a self‐powered stimulation system in vivo has great potential.^[^ ^27^ ^]^ The biggest advantage is that the defects of implantable battery replacement can be avoided, and the amount of electrical stimulation is more stable and larger than that of biofuel cells.^[^ ^28^ ^]^

#### 2.1.4 Wireless electrical stimulation

**FIGURE 2:**
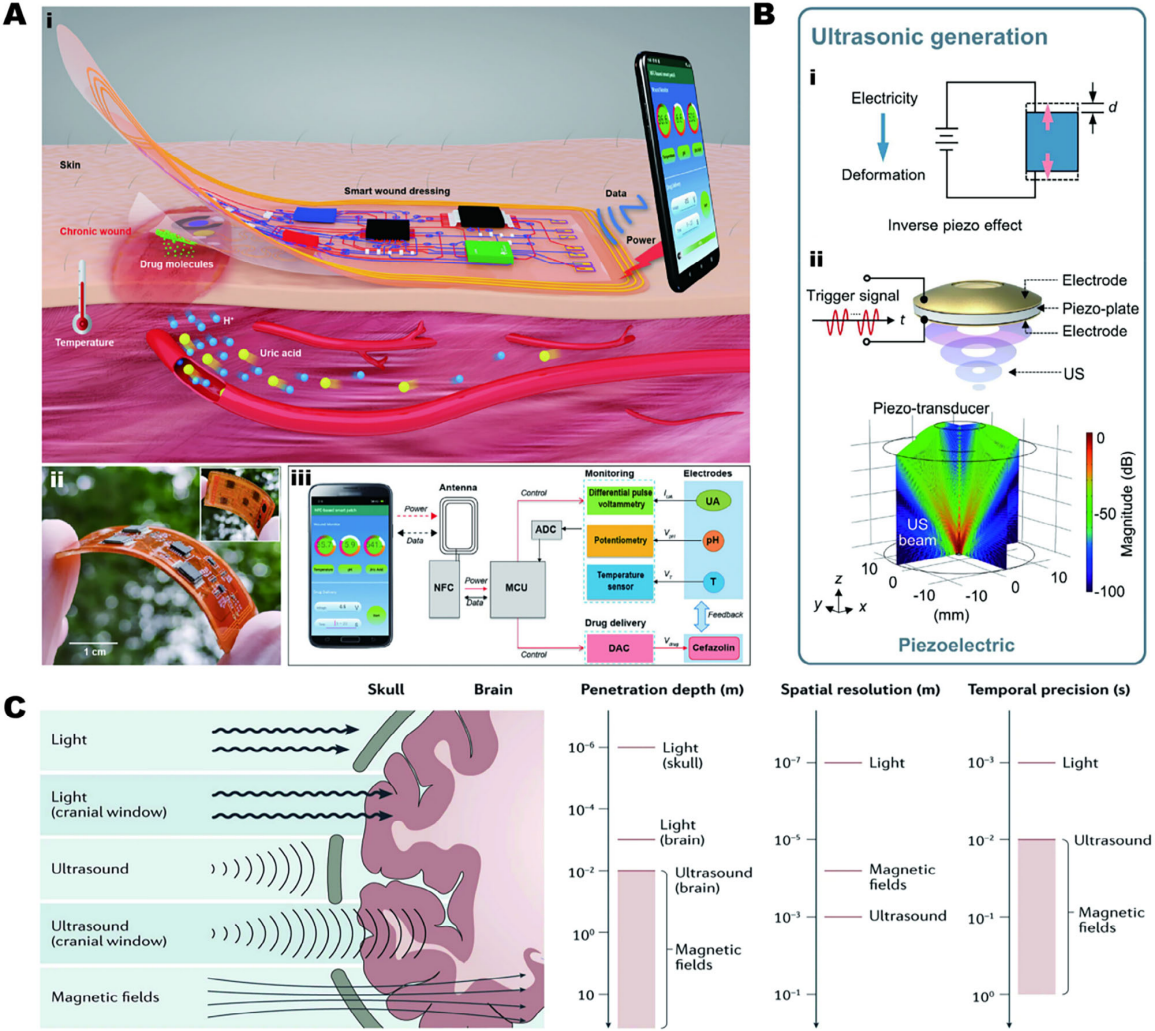
(A) Wireless electrical stimulation system. (i) Schematic diagram of wireless electrical stimulation; (ii) Electrode layer hidden under circuit layer; (iii) Working principle of the system. Reproduced with permission.^[^ ^29^ ^]^ Copyright 2021, John Wiley & Sons. (B) Principle of ultrasonic generation based on inverse piezoelectric effect: (i) Schematic diagram of the inverse piezoelectric effect and (ii) finite element simulation of the sound field of piezoelectric US transducer. Reproduced with permission.^[^ ^37^ ^]^ Copyright 2023, Elsevier. (C) Schematic diagram of tissue penetration by light stimulation, ultrasonic stimulation, and magnetic stimulation. Reproduced with permission.^[^ ^11^ ^]^ Copyright 2021, Elsevier.

The wireless electrical stimulation system allows programmable electrical stimulation by implanting tiny and light‐weight devices in the body, again avoiding percutaneous wires and bulky power supply units. Compared with implanted electrical stimulation, it has higher controllability and greater accuracy. It is an ideal electrical stimulation strategy, as shown in Figure 2A.^[^ ^29^ ^]^

### 2.2 Light stimulation

Ultraviolet, visible, and near‐infrared light exposure is known as low‐level laser therapy and is also known as photobiomodulation therapy.^[^ ^30^ ^]^

When seeking to develop photoresponsive biomaterials, it should be considered that material handling during synthesis and utilization, including through processing in dark research spaces, can minimize premature photoactivation of low‐energy photosensitive materials. In a variety of wavelengths of light, near‐infrared light is abundant across the entire range of the solar spectrum and damages negligibly tissue, a unique function that makes near‐infrared light‐responsive materials ideal candidates for therapeutic agents and biological tools for biomedical applications.^[^ ^31^ ^]^ However, the low energy endowed by near‐infrared light also limits the high‐energy reaction. Visible light is more for the application of photocatalytic hydrolysis‐related directions, simulating the catalytic application in the natural environment of natural light.^[^ ^32^ ^]^ Near ultraviolet light and high‐energy ultraviolet light can cause a lot of damage to the surface of the material.^[^ ^33^ ^]^ For the development of functional azo‐based materials, the switch must be triggered by ultraviolet light, which greatly damages the corresponding surface, but the high scattering and weak penetration of short‐wavelength ultraviolet light are very limited in application.^[^ ^34^ ^]^ And long‐term exposure to ultraviolet light may be carcinogenic.^[^ ^35^ ^]^ Although ultraviolet light is more commonly used in sterilization, the choice of ultraviolet light needs to be more careful based on the theory that risks must be balanced with beneficial effects. Light remains a uniquely powerful stimulus that regulates the properties of dynamic biomaterials. Continued innovation in the synthesis of photosensitive biomaterials in response to low‐energy light will lead to increased opportunities for activation in vivo of complex tissues and biologically relevant sites. Future opportunities for the conversion of this light‐sensitive material remain bright.^[^ ^36^ ^]^

### 2.3 Ultrasonic stimulation

Ultrasonic stimulation can be divided into low‐intensity ultrasound and high‐intensity ultrasound. When piezoelectric materials are subjected to an electric field applied along the direction of their polarization, they deform. Once the electric field is removed, the deformation will disappear. This phenomenon is known as the inverse piezoelectric effect, as shown in Figure 2B. When a periodic alternating current field is applied to the piezoelectric plate, the corresponding periodic deformation produces vibration, which is propagated in the acoustic medium in the form of sound waves.^[^ ^37^ ^]^ For low‐intensity ultrasound, diagnostic and repair functions dominate because the periodic mechanical waves, which are inaudible to humans, are controllable, non‐invasive, and highly penetrating to tissues. For high‐intensity ultrasound, heating effects and sound cavitation dominate, meaning that the cells are destroyed. Therefore, high‐intensity ultrasound is mainly used for killing cancer and removing bacteria.^[^ ^38^ ^]^ In addition, the boundary between high‐intensity and low‐intensity ultrasound is not clear.^[^ ^39^ ^]^

### 2.4 Magnetic stimulation

Magnetic stimulation is regarded as the key to achieving non‐invasive stimulation, and accurate, controlled release has been widely used in tissue engineering therapy.^[^ ^40^ ^]^ It is worth noting that the mediation of magnetic stimulation on tissue mainly depends on frequency, duration, and intensity. In addition, whether it is different scales in the tissue, or different patients, the parameters that regulate magnetic stimulation can provide customized treatment.^[^ ^41^ ^]^ The therapeutic effect of magnetic stimulation alone is limited. Combining magnetic stimulation with other strategies to synergistically promote tissue repair is a promising strategy. At the same time, compared with electricity, light, and ultrasound, magnetic stimulation has good penetration ability, which is also used for deep treatment, as shown in Figure 2C.^[^ ^11^ ^]^ In conclusion, magnetic stimulation has a broad application prospect as an adjunct therapy for tissue repair.

## 3 RESPONSIVE BIOMATERIALS

From the perspective of the historical process of human development, biological materials have made great contributions to the healthy lives of human beings, laying the foundation for the further development of modern bioengineering. With the continuous progress of science and technology and the continuous improvement of medical living standards, intelligent response biomaterials have attracted more and more researchers' attention.

### 3.1 Electrical responsive biomaterials

In general, electrical‐responsive e‐biomaterials are composed of electroactive biomaterials consisting mainly of organic biomaterials such as (polypyrrole (PPy), poly(3,4‐ethylenedioxythiophene) (PEDOT), poly(aniline) macromolecule (PANI)^[^ ^42^, ^43^, ^44^ ^]^), inorganic biomaterials (carbon nanotubes, reduced graphene oxide, graphene oxide^[^ ^45^, ^46^, ^47^ ^]^), novel two‐dimensional biomaterials (MXene, black phosphorus),^[^ ^48^, ^49^ ^]^ and the common preparation methods include hydrothermal method, high temperature sintering, oxidation‐reduction method, and so on. Electrical‐responsive biomaterials not only provide a growth scaffold for cells but also reduce the current loss caused by the surrounding medium. Electrical‐responsive biomaterials can adjust the biochemical and biophysical reactions of their materials through external electrical stimulation, including adjusting the mechanical properties of materials, redox state, hydrophobic/hydrophilic energy, surface ligand conformation, and so on, to build different microenvironmental conditions for cells or surrounding tissues. Electro‐induced mechanical force or electrical force responsive to the movement of biomaterials can also be used for targeted therapy and controlled drug release.^[^ ^50^ ^]^ At the same time, it can also affect cells, including the control of cell adhesion, migration, and stem cell differentiation by electrically inducing cell Arg‐Gly‐Asp (RGD) conformational changes,^[^ ^51^, ^52^ ^]^ cell membrane deformation, ion channel activation, integrin‐mediated cytoskeleton remodeling and so on.^[^ ^53^ ^]^ Therefore, electrical stimulation based on electrical‐responsive biomaterials can provide a diversified platform for the application of tissue engineering and intelligent response therapy in biomedicine.

### 3.2 Ultrasonic responsive biomaterials

When ultrasonic waves travel through human tissues and are used on biological materials, there are physical effects that trigger ultrasound‐mediated biological responses. The ultrasonic response properties of biomaterials are influenced by a variety of factors, including the composition and shape of the biomaterial and, most importantly, the ability to convert sound energy into more useful forms required for tasks such as triggering drug release or mechanically initiating biological processes. It is mainly divided into inorganic nanoparticles,^[^ ^54^, ^55^, ^56^ ^]^ liposome,^[^ ^57^, ^58^, ^59^ ^]^ microbubbles or nanobubbles,^[^ ^60^, ^61^, ^62^ ^]^ and so on.

The most studied principles of its response are the piezoelectric and cavitation phenomena. Piezoelectric nanogenerators based on non‐centrosymmetric crystal materials deform under external mechanical forces to induce potential differences, so when sound waves act on the material, internal polarization occurs and energy conversion is completed (sound energy is converted to electrical energy). This change in properties is a widely used method in fields such as anti‐infection and osteogenesis, which determines the ultrasonic response of the material to mechanical waves.^[^ ^63^ ^]^

Cavitation is an important mechanism of ultrasonic transducers. In this process, sound waves are set to oscillate, causing bubbles to form and collapse. Due to the strong response of the bubbles to ultrasonic waves, a large amount of energy is converted into the movement of the bubbles, thus providing powerful mechanical and thermal stimulation. The plasma formed during the bursting of bubbles plays an important role in chemistry. Based on this, some bubble‐like nanomaterials can also produce a strong response to ultrasonic waves through the cavitation effect. Drug release can be achieved through ultrasound‐induced cavitation.^[^ ^64^ ^]^ Which is a common method of controlling release.

### 3.3 Light responsive biomaterials

Common light responsive biomaterials are mainly divided into two kinds. One is the photothermal material. The ideal photothermal material should have a strong absorption capacity in the near‐infrared region, and can effectively convert the absorbed light energy into heat energy. Mainly some metal‐based materials and 2D nanomaterials. Due to the strong surface plasmon resonance effect of metal‐based materials, the free electrons of metals can coherently oscillate around the surface of nanoparticles by using laser irradiation resonant with the frequency absorbed by the nanomaterial, and the non‐radiative extinction caused by absorption and scattering can be effectively converted into local heat by picosecond electron–electron and electron–phonon relaxation.^[^ ^65^ ^]^ Considering that metal nanomaterials have a plasmon resonance band in the near‐infrared region is ideal, the factors affecting the surface charge density can be appropriately modified, such as transforming the size, shape, structure, and dielectric properties of the metal and the surrounding medium to achieve the purpose.^[^ ^66^ ^]^ At the same time, metal‐based materials also have good synthesis adjustability, so some metal nanomaterials are considered to be simple and effective photothermal reagents with superior optical and photothermal properties.^[^ ^67^ ^]^ In addition to metal photothermal agents, some 2D nanomaterials are also candidates with good photothermal effects because they have significant plasma effects on near‐infrared light irradiation. Compared with other nanomaterials, two‐dimensional nanomaterials have the advantages of atomic layer structure, controllable optical properties, easy preparation, a large specific surface area, and convenient functionalization.^[^ ^68^ ^]^

The second type is light reactive materials. One of them, known as the photodynamic effect, changes from a steady state to an excited state when the material is irradiated by light energy. However, the excited state is unstable and will react in a steady‐state direction, in which a large number of reactive oxygen species will be produced.^[^ ^69^, ^70^ ^]^ However, the photodynamic effect of this photocatalysis is limited by the rapid charge recombination and the limited use of exogenous stimulation of the catalyst, so it is necessary to fully consider the selection of material properties and combinations, similar to some special heterojunction structures with extremely high photocatalytic efficiency.^[^ ^71^ ^]^ In addition, photoresponsive hydrogels are a very important photoreactive biomaterial, including photocrosslinking, photodegradation, etc. The micro‐scale structure of hydrogels is controlled by light stimulation to regulate their physical and chemical properties, which has also achieved rapid development in recent years.^[^ ^72^, ^73^ ^]^ Among them, hydrothermal in‐situ synthesis, hydrogen bonding, electrostatic adsorption, chemical bond breaking, and recombination are the most common synthesis methods.

### 3.4 Magnetically responsive biomaterials

Magnetically responsive biomaterials are composed of magnetically active materials and biomaterials, and common magnetically active materials include iron oxide (Fe_3_O_4_^[^ ^74^ ^]^ and Fe_2_O_3_^[^ ^75^ ^]^), transition metal ferrite (CoFe_2_O_4_^[^ ^76^ ^]^ and MnFe_2_O_4_^[^ ^77^ ^]^) and transition metal alloys,^[^ ^78^ ^]^ which are also widely used in biomedicine. Usually, because the magnetically active material is prepared in the organic phase, early surface modifications are aimed at improving the biocompatibility and chemical stability of the magnetically active material. With the deepening of research, more advanced modification methods for the surface of magnetic active materials are emerging endlessly, and more functional designs are also being brought in.

## 4 APPLICATION OF BIOMATERIALS IN RESPONSE TO PHYSICAL STIMULATION

**FIGURE 3:**
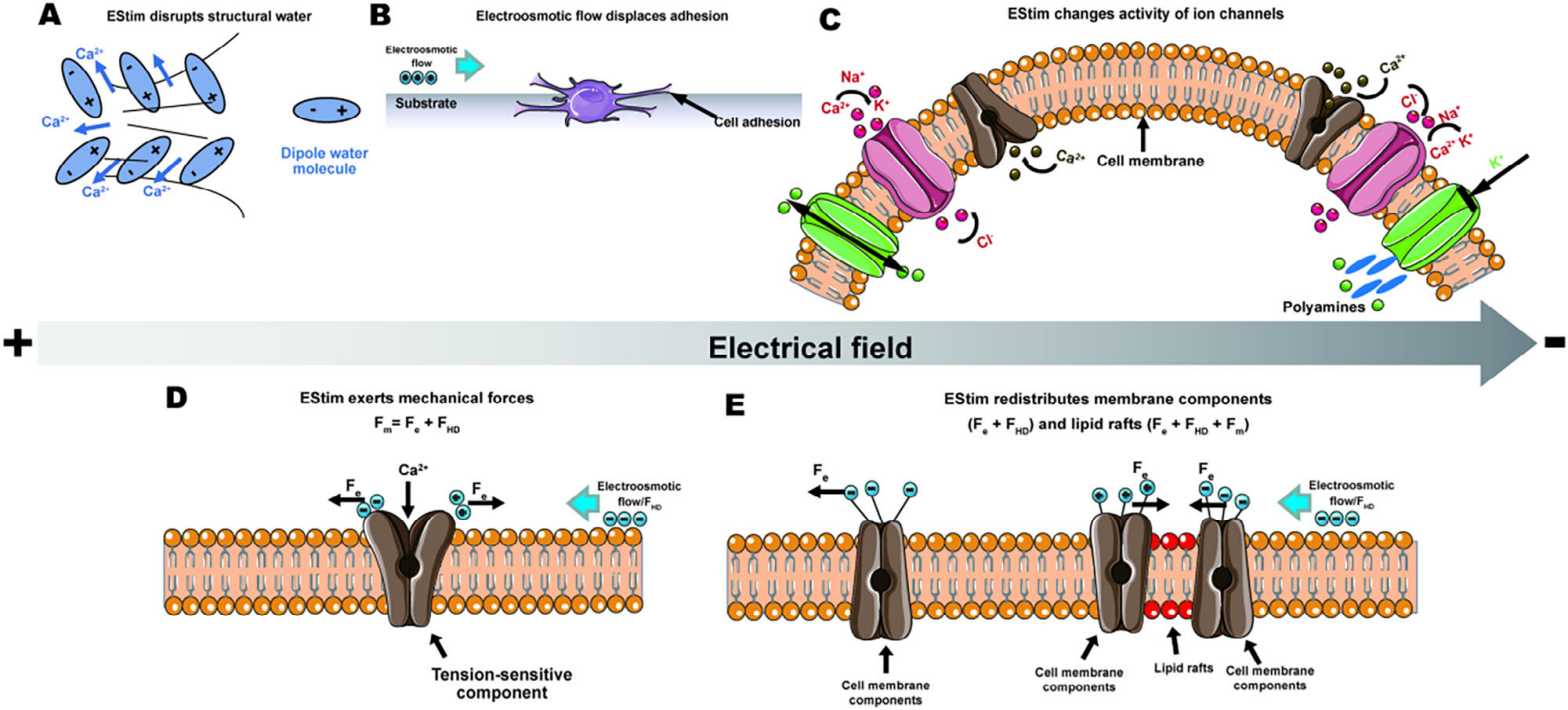
Hypothesis of cell response to electrical stimulation. (A) Structural water damage. (B) Volume flow of electro seepage. (C) Asymmetric ion flow and voltage‐gated channel opening. (D) Mechanical sensation. (E) Redistribution of membrane composition and lipid rafts. Reproduced with permission.^[^ ^1^ ^]^ Copyright 2020, Elsevier.

The basic physical mechanism of cell response to electrical stimulation is currently under active investigation. A few hypotheses have been proposed and are summarized below: (1) structural water failure; (2) electro‐seepage volume flow; (3) asymmetric ion flow and voltage‐gated channel opening; (4) mechanical sensation; (5) redistribution of membrane components and lipid rafts,^[^ ^1^ ^]^ such as in Figure 3. The proposed basic principle and mechanism provide theoretical support for the application of electrical‐responsive biomaterials.

### 4.1 Application of biomaterials in response to electrical stimulation

#### 4.1.1 Electrical stimulation response biomaterials for neural repair

**FIGURE 4:**
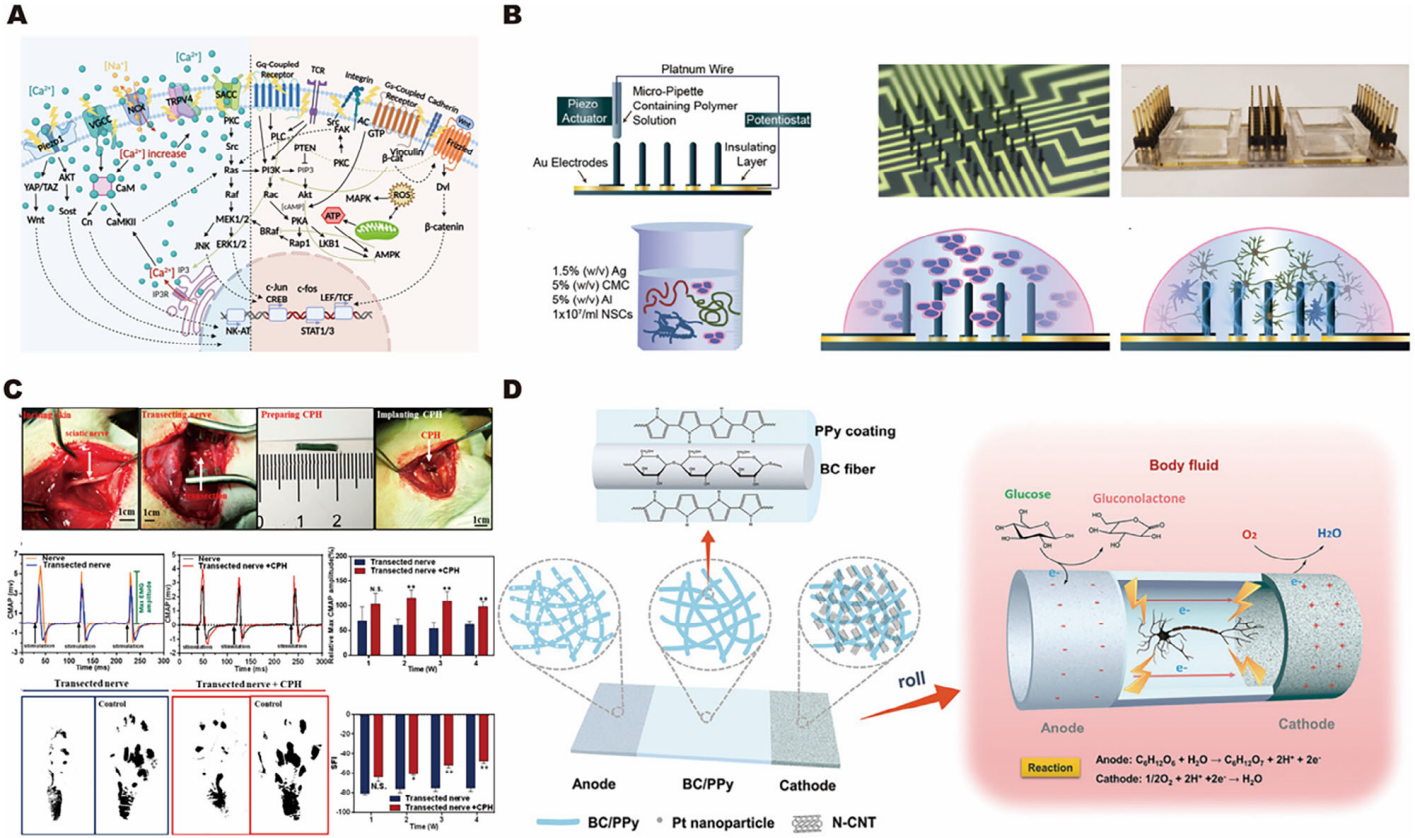
Application of electrical stimulation‐response biomaterials for nerve repair. (A) Cell‐level mechanism of electrical stimulation promoting nerve repair. Reproduced with permission.^[^ ^81^ ^]^ Copyright 2023, Elsevier. (B) 3D culture of electrical stimulation‐response to nerve regeneration. Reproduced with permission.^[^ ^84^ ^]^ Copyright 2019, John Wiley & Sons. (C) Neural injury model and repair. Reproduced with permission.^[^ ^89^ ^]^ Copyright 2020, American Chemical Society. (D) Biobattery electrical stimulation system. Reproduced with permission.^[^ ^42^ ^]^ Copyright 2019, John Wiley & Sons.

Autologous or allogeneic nerve transplantation is the traditional method for nerve injury repair.^[^ ^79^ ^]^ However, due to the limited number of donors and the incomplete recovery function, the clinical therapeutic effect is not ideal.^[^ ^80^ ^]^ Compared with the central nervous tissue, the stronger reproducibility of peripheral nervous tissue enables the regeneration and repair of damaged and broken peripheral nervous tissue to get rid of traditional surgical treatment.^[^ ^81^ ^]^ However, the regeneration of nerve tissue is limited, and the complex and uncontrollable microenvironment makes the clinical application of peripheral nerve regeneration difficult, especially for long‐distance nerve tissue repair (larger than 5 cm), the possibility of self‐regeneration in the natural environment is very small.^[^ ^82^ ^]^ As the most common means of peripheral nerve repair, electrical stimulation can accelerate the transfer to the injured site for wound repair by improving the signaling pathways related to the regeneration of the lower nerve under the action of the electric field, including the regeneration factors, receptors and related proteins, and endogenous reactions, as shown in Figure 4A.^[^ ^83^ ^]^

A large number of studies on long‐distance neural tissue repair have focused on the construction of neural‐guided catheter models using electrical stimulation response materials,^[^ ^84^ ^]^ so high biosafety and high electrical stimulation response abilities are particularly important. Huang et al. prepared a composite hydrogel by combining modified chitin with PEDOT electrostatic force.^[^ ^85^ ^]^ The active site on the polypeptide‐modified surface also increased the proliferation and adhesion of Schwann cells, the main role of nerve regeneration, and the expression of photogenic genes and proteins. However, the neural guide catheter prepared by this method is limited to 2D cell culture and cannot truly simulate the real cell growth microenvironment in natural human tissues and organs. Therefore, Tomaskovic–Croo et al. constructed a neural catheter with a 3D cell microenvironment by using 3D tissue engineering technology.^[^ ^86^ ^]^ The stem cells were encapsulated in a 3D gel made of alginate, chitosan, agar, and a microelectrode array was added. Poly(3,4‐ethylenedioxythiophene)/ poly(styrenesulfonate) (PEDOT: PSS) electrical stimulation response coating was prepared at the same time, as shown in Figure 4B. It greatly aided the ion migration and differentiation of neural stem cells. Scientists are also committed to the study of nerve conduits for spontaneous electrical stimulation. The frozen gel of fibroin protein prepared by Ma et al. by gradient freezing uses a porous surface to bind PEDOT in situ, and uses the piezoelectric properties of poly (vinylidene fluoride) (PVDF) outside to transform its nerve catheter into spontaneous electrical stimulation under the action of mechanical force.^[^ ^87^ ^]^ In addition, to increase its biodegradability, poly(l‐lactide‐*co*‐caprolactone) (PLCL) is added without reducing its piezoelectric ability. It is endowed with good biodegradation ability and is committed to not carrying out secondary trauma to the wound site. At the same time, the multi‐channel bionic structure constructed by the authors is also conducive to limiting the diffusion of axons and preventing the wrong branch of axons. As a common material in response to electrical stimulation, PEDOT's poor biodegradation ability also limits the development of its clinical application. Guarino et al. prepared PANI in situ in a polyethylene glycol diacrylate solution, then made it into a gel by ultraviolet crosslinking and made a porous structure by leaching sodium chloride particles to ensure the response‐ability of PANI to cells after electrical stimulation.^[^ ^88^ ^]^ Due to the absence of more internal chemical links, the mechanical properties of the gel prepared by this method are poor, and the PANI fiber film alone has poor mechanical strength.^[^ ^89^ ^]^ Thus, to meet the basic mechanical requirements of nerve conduits, researchers need to introduce more connections, such as hydrogen bonds, into the system. Xu et al. synthesized PANI in situ from cellulose hydrogel by a limited interfacial polymerization method.^[^ ^90^ ^]^ However, this simple synthesis could not effectively fix PANI in the gel, so hydrogen bonds in phytic acid were used to fix PANI in the gel to enhance its mechanical properties. The amide bond energy of poly(acrylamide) (PAM) interacts with PANI to form a hydrogel with excellent mechanical properties, which is a prerequisite for nerve conduit. Not only do PAM and PANI have an electrical stimulation response, but the hydrogel mixed with PAM and PANI could also respond to near‐infrared light, and its conductive ability can also become stronger with the extension of irradiation time. The peripheral nerve injury model was constructed, and it was found that the presence of nerve conduits could replace the damaged nerves to achieve the transmission of bioelectrical signals and the recovery of secondary functions, as shown in Figure 4C.^[^ ^91^ ^]^

PPy has the same excellent biocompatibility and electrical conductivity as PEDOT and PANI. As an electrospinning film, it not only lacks mechanical properties but also biodegradability. Therefore, using the biodegradability of poly(lactic‐*co*‐glycolic acid) (PLGA) and the good mechanical properties of polycaprolactone (PCL) to modify it is expected to achieve the best degradation rate.^[^ ^92^ ^]^ To prevent the disadvantages of secondary trauma and the increased risk of infection caused by external electrical stimulation, piezoelectricity, and trigeneration have been extensively studied as common means of self‐generating electricity. The self‐powered neural conduit of glucose and oxygen consumption in vivo is a relatively new research direction. Sun et al. used PPy to form a conductive substrate by in‐situ polymerization on bacterial cellulose and deposited platinum nanoparticles on the anode for glucose oxidation.^[^ ^42^ ^]^ The cathode was loaded with nitrogen‐doped carbon nanotubes, as shown in Figure 4D. It is certain that more research is needed in the future to explore the direction of self‐powered electrical stimulation.

#### 4.1.2 Electrical stimulation response biomaterials for skin repair

Wound repair is mainly divided into epidermal skin wound repair and internal organ wound repair.^[^ ^93^ ^]^ Due to the high controllability of physical stimulation on the surface and the advantages of visualization, most wound repair research focuses on skin wound repair. During the period of skin damage, external stimulation is more likely to directly harm the body itself. The faster rate of skin repair means the establishment of a safer human protective barrier, however, due to the stress of the wound site or the problems of internal diseases such as diabetes or venous skin ulcers.^[^ ^94^ ^]^ The rate of skin repair is greatly reduced. At the same time, when the epidermal barrier is damaged for a long time, the wound will be further infected by bacteria to form a biofilm. The wound is then unable to exit the inflammatory phase, so the wound cells become senescent and less responsive to stimulation.^[^ ^95^ ^]^ Therefore, the use of external stimulation to accelerate the healing of skin wounds is extremely important. Skin wound healing mainly involves granulation, epithelialization and vascularization, hair follicles, and inflammatory and immune responses.^[^ ^96^ ^]^ Condition optimization for the involved reactions provides a positive driver for promoting skin wound healing.

**FIGURE 5:**
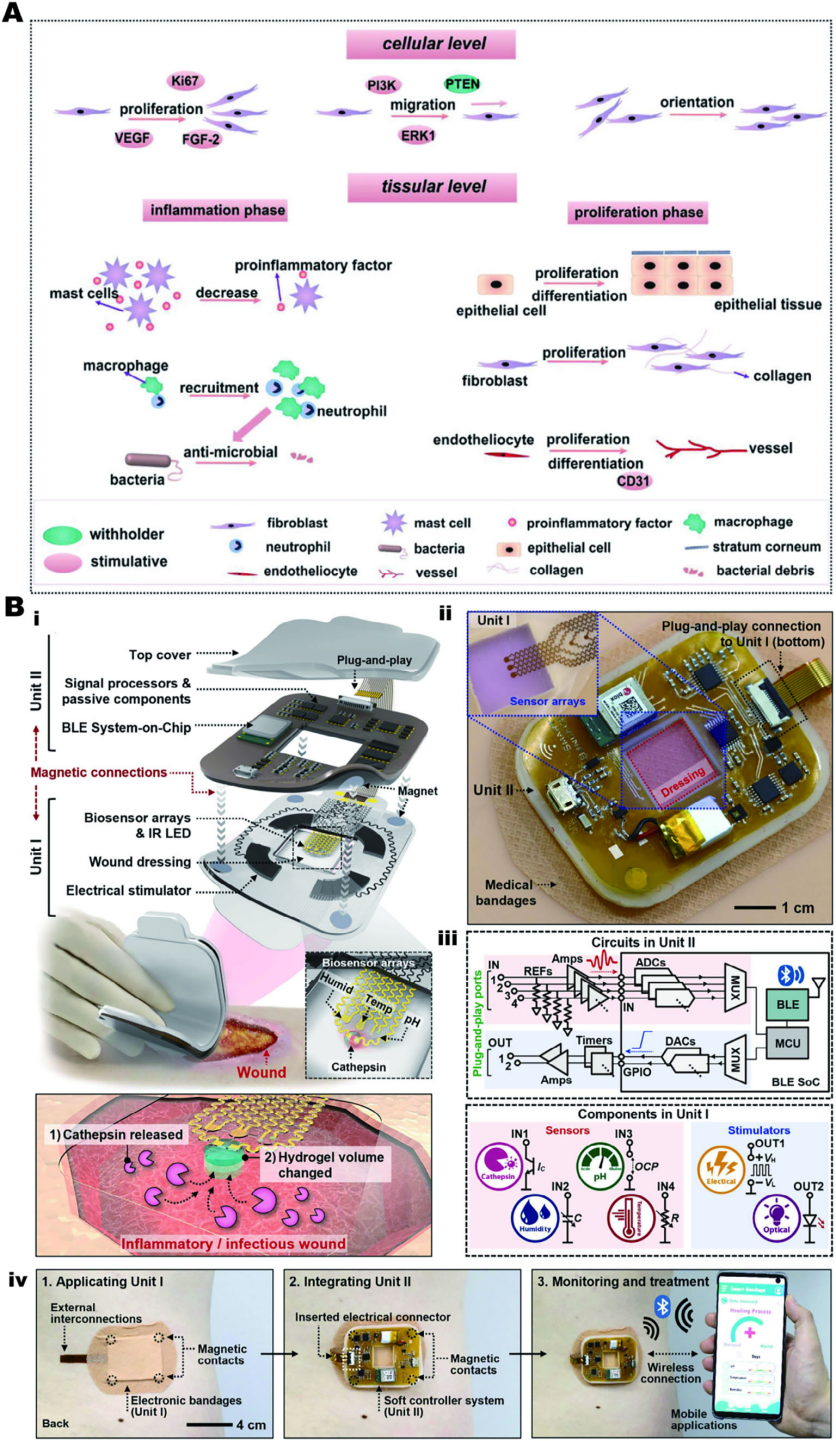
Application of electrical stimulation in response to biomaterials in skin repair. (A) Electrical stimulation promotes improvement in skin repair cell levels. Reproduced with permission.^[^ ^92^ ^]^ Copyright 2021, John Wiley & Sons. (B) Visual monitoring of skin repair by electrical stimulation. (i) Electronic bandage system; (ii) enlarged view of the entire system; (iii) operational flow chart of the electronic system; (iv) electronic bandage system application sequence. Reproduced with permission.^[^ ^13^ ^]^ Copyright 2022, Elsevier.

Some studies have proved that the construction of an appropriate amount of electrical stimulation in vitro can improve the wound microenvironment, promote the proliferation and differentiation of key cells at the cellular level, and accelerate migration.^[^ ^97^, ^98^, ^99^ ^]^ At the tissue level, the expression of inflammatory factors in the inflammatory period and the promotion of the proliferation and differentiation of skin cells during the proliferation period can promote wound healing, as shown in Figure 5A.^[^ ^94^ ^]^ However, for the complicated equipment of external electrical stimulation and the limited range of motion, the integrated equipment of the electrical stimulation device and wound dressing is more flexible and convenient. Using polymer piezoelectric materials such as poly(l‐lactide) (PLLA),^[^ ^100^ ^]^ and PVDF^[^ ^101^ ^]^ can enable the prepared material to have the ability of spontaneous electrical stimulation. Of course, because many metals and their oxides have excellent electrical conductivity and piezoelectric ability, they are often added to the construction of self‐electric equipment, and metal and their oxides slowly release metal ions or produce reactive oxygen species during electrical stimulation, which also gives them certain antibacterial and anti‐infection ability, reducing the occurrence of inflammation and accelerating the healing of wounds.^[^ ^102^, ^103^ ^]^ In addition, the presence of a large number of free borax ions in borax hydrogels will trigger the ionic conductive properties of the material to respond to the stimulation of current signals, which can not only promote wound healing but also reduce bacterial infection to a certain extent.^[^ ^104^ ^]^

At the same time, the concept of further real‐time monitoring of wounds based on wound recovery in response to electrical stimulation will also have a place in future visual medicine.^[^ ^105^ ^]^ Yang et al. have prepared a flexible electronic dressing that can be monitored in real‐time and respond to light and electrical stimulation at any time based on the detection of biochemical markers of inflammation, as shown in Figure 5B, and proved its feasibility through a mouse model.^[^ ^13^ ^]^

#### 4.1.3 Electrical stimulation response biomaterials for bone repair

Piezoelectricity is one of the inherent characteristics of natural bone, mainly due to the piezoelectric effect of collagen molecules.^[^ ^106^ ^]^ However, a bone tissue defect or injury under the condition of disease or trauma will reduce the potential level of the injured site, which will affect the normal bioelectrical transmission of the body.^[^ ^107^ ^]^ Researchers have found that, in addition to endogenous electron fields, the electric field generated by external electrical stimulation is also a key factor in regulating metabolic activities such as fracture healing and structural remodeling.^[^ ^108^, ^109^ ^]^ Therefore, based on the electrical cues of natural bone, researchers are focusing on the development of bone tissue repair assisted by electrical stimulation in response to biological materials.^[^ ^110^, ^111^ ^]^

**FIGURE 6:**
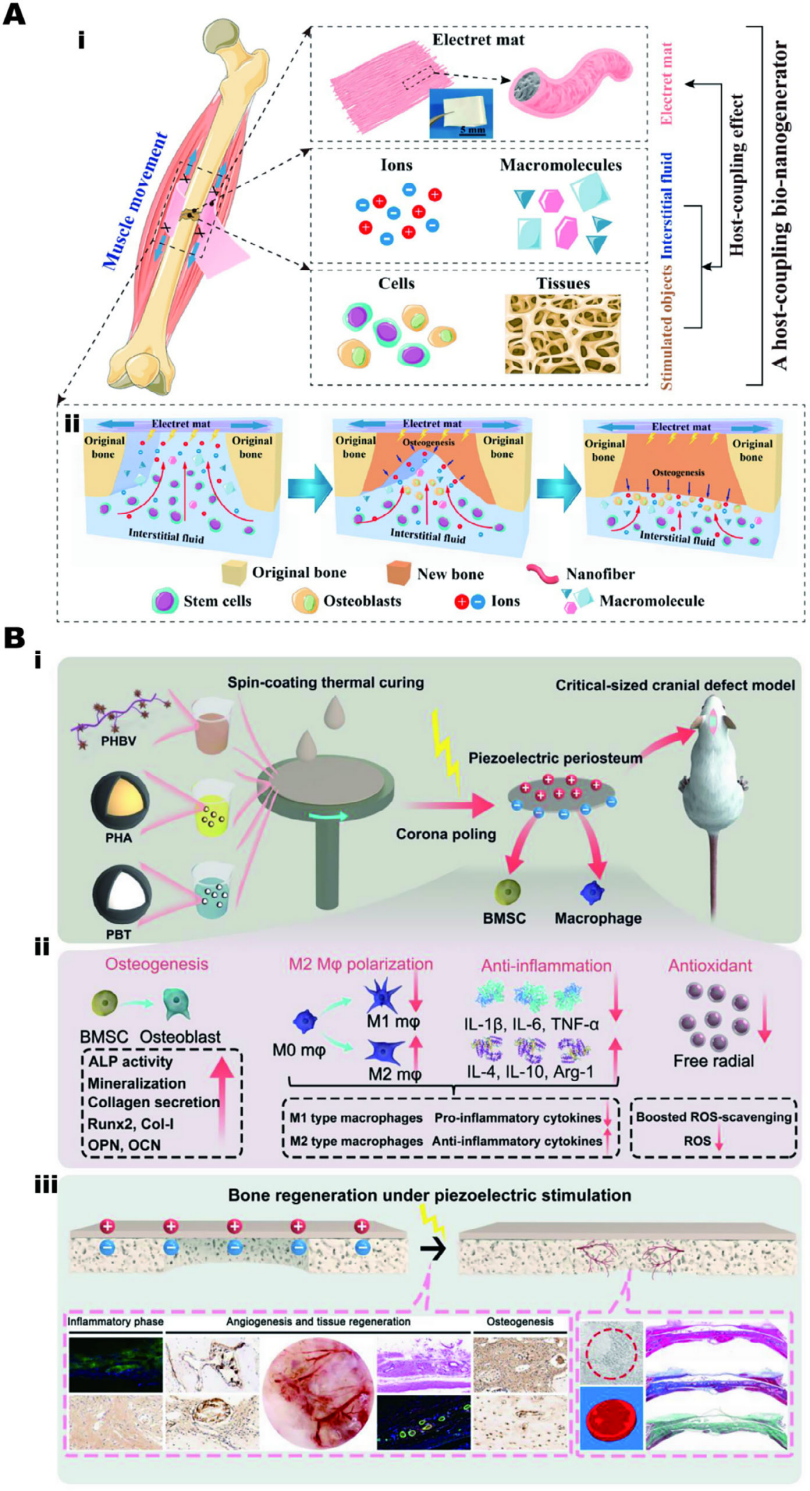
Application of electrical stimulation‐response biomaterial for bone repair. (A) The electrical stimulation response regulates related proteins and enzymes to accelerate bone repair. (i) Schematic diagram of an electret‐based host‐coupling bio‐nanogenerator implanted onto the bone injury in vivo. (ii) Mechanism of bone repair. Reproduced with permission.^[^ ^113^ ^]^ Copyright 2021, Elsevier. (B) Preparation of electrical stimulation response system (i). Accelerates stem cell differentiation, regulates immunophenotype, reduces inflammatory expression, and reduces reactive oxygen species accumulation (ii). Accelerates bone repair (iii). Reproduced with permission.^[^ ^114^ ^]^ Copyright 2023, American Chemical Society.

The potential difference exists inside and outside the cell for ion transport. Changes in ion concentration inside and outside the cell membrane in the presence of external field electrical stimulation, especially Ca^2+^ ions, will lead to changes in cell function.^[^ ^112^ ^]^ Due to their natural negative charge, the cells will also move toward the injured site and compact under the action of the electric field to accelerate bone tissue repair.^[^ ^113^ ^]^ The time and strength of current stimulation have a great influence on bone tissue repair. Long‐term electrical stimulation may make the current unstable, and the effect of charge polarity on osteogenic differentiation is difficult to determine. However, heterostructures with good polarization performance can effectively improve such a situation.^[^ ^114^ ^]^ Appropriate electrical stimulation response material can activate calmodulin, calcineurin, and NFAT pathway up‐regulation in vivo and enhance the osteogenic differentiation ability of stem cells, as Figure 6A.^[^ ^115^ ^]^ Researchers also believe that appropriate electrical response materials that can transmit electrical signals can not only promote the directed differentiation of stem cells into osteoblasts but also play a role of directed selection in immune regulation, promote the M2 process and reduce the M1 process, and to a certain extent reduce the expression of inflammatory factors and enhance the resistance of cells to oxidative stress, as shown in Figure 6B.^[^ ^116^ ^]^

**FIGURE 7:**
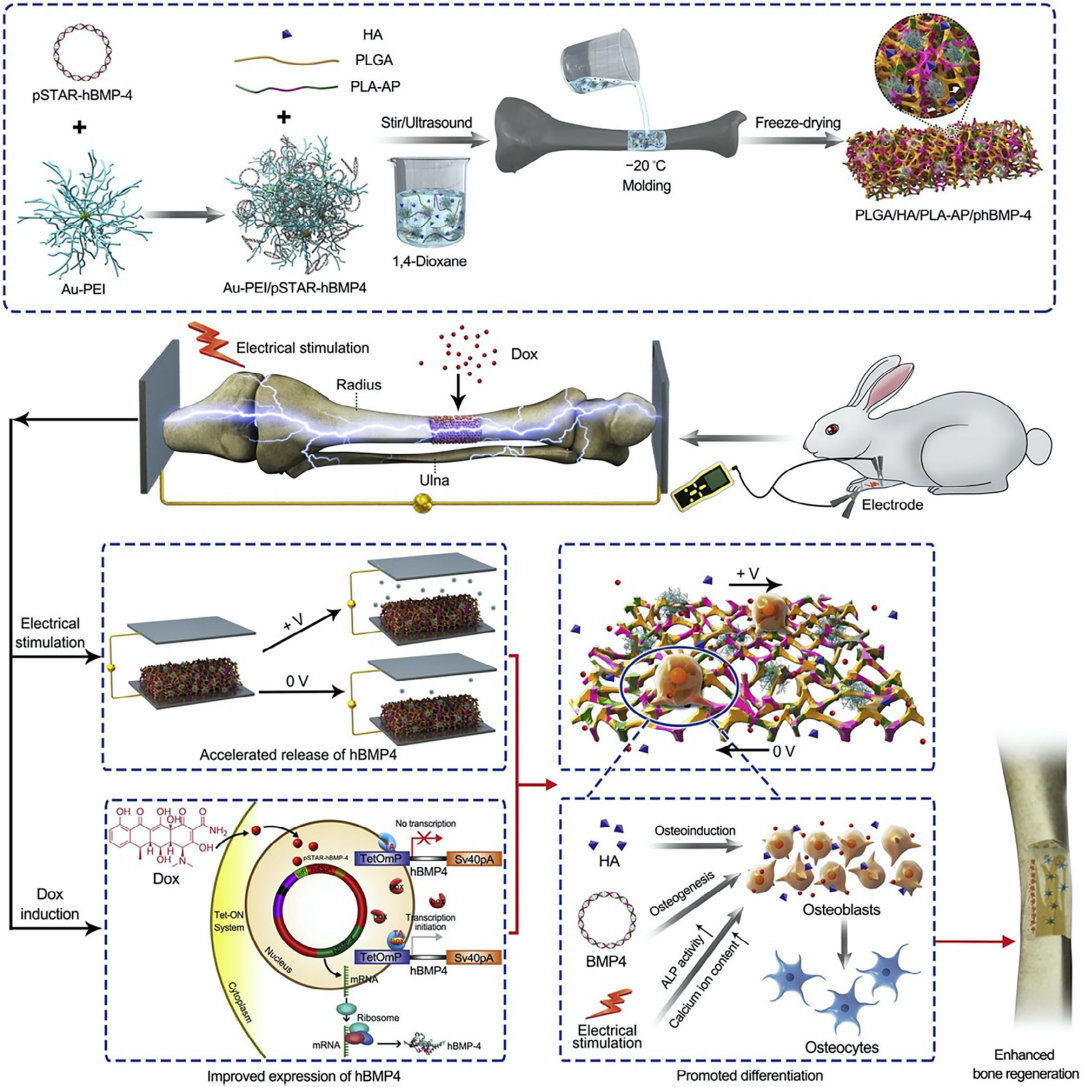
Application of electrical stimulation‐response biomaterial for bone repair. Controlled release of growth factors to accelerate bone tissue regeneration. Reproduced with permission.^[^ ^14^ ^]^ Copyright 2020, Elsevier.

Although a single electrical stimulation response material has shown good therapeutic effects in bone tissue engineering, the multi‐angle composite collaborative repair will show a faster, more flexible, and more comprehensive application. For example, the load of growth factor hBMP‐4, under current stimulation not only enables the electro‐responsive material to transmit electrical stimulation signals but also controls the release of hBMP‐4, induces the formation of bone collagen, promotes the differentiation of osteoblasts, and reduces the formation of scar tissue trauma, as shown in Figure 7.^[^ ^14^ ^]^

### 4.2 Application of biomaterials in response to light stimulation

#### 4.2.1 Light‐responsive biomaterials photothermal and photodynamic

Due to the limited light penetration ability, it is difficult to carry out deep stimulation in the body, so the main application scenarios of biological materials with light response are in the surface tissue, and their biggest advantage is convenient operation and easy control. Photothermal and photodynamic are the most common behaviors of photoresponsive biomaterials and are widely used in tissue repair due to their excellent biosafety and good antibacterial and antitumor activities.^[^ ^117^, ^118^ ^]^ However, a single photothermal or photodynamic process requires a higher temperature or the production of more reactive oxygen species to play an effective repair role. It may cause great side effects to the normal body. Therefore, more researchers choose the synergistic treatment of photothermal and photodynamic for rapid repair.^[^ ^119^ ^]^ Local temperature increases in collaborative therapy can enhance the membrane permeability of bacteria or tumor cells and are more conducive to reactive oxygen species invasion and disruption of internal homeostasis. According to the latest research progress, photoresponsive biomaterials are mainly classified into metal compounds,^[^ ^120^, ^121^, ^122^ ^]^ polydopamine groups,^[^ ^123^, ^124^, ^125^ ^]^ carbon‐based materials,^[^ ^126^, ^127^, ^128^ ^]^ and so on.

Metallic compounds are widely distributed and prepared by various methods and have been proven to be promising photothermal agents. Among them, copper sulfide nanoparticles (CuS NPs), as the most common metal sulfide photoresponsive particles, are P‐type semiconductor materials with excellent photothermal and photodynamic catalytic properties, low biotoxicity, and a large near‐infrared absorption range. Researchers have used various means to disperse it in various material carriers to exert its photo response‐ability, and it has been applied in skin repair.^[^ ^129^, ^130^ ^]^ With the continuous deepening of research on photoresponsive materials, more non‐metallic photoresponsive materials have been developed. Compared with the photoresponsive metal oxides and their sulfide, non‐metallic photoresponsive materials greatly avoid the release of harmful metal ions and reduce the risk of biological toxicity. Carbon‐based materials have high thermal stability, and long‐term laser irradiation will not cause performance attenuation. They not only have strong light absorption in the visible light to near‐infrared region but can also interact with biomolecules, cells, and even natural tissues, which can not only achieve antibacterial ability but also promote skin wound regeneration.^[^ ^131^ ^]^ Carbon nanotubes benefit from major biocompatibility and processability, and their preparation into different forms will benefit the healing of different skin wounds. Sun et al. prepared the regular microneedles into a microneedle patch. The regular array of microneedles can guide the directional growth of fibroblasts, and the photoresponse ability of carbon nanotubes can control the microneedle to release corresponding drugs to assist wound treatment.^[^ ^132^ ^]^ Graphene oxide's excellent light response and ability to attach high nanomaterials due to its high specific surface area make it widely used in skin repair.^[^ ^133^, ^134^ ^]^ Due to its high electronegativity, graphene oxide can also form complexes by condensation with cationic polymers through electrostatic interactions. Guo et al. used this property to bind it to electropositive chitosan quaternary ammonium salt to control drug release through a photothermal response, thus achieving the effect of eliminating bacteria.^[^ ^135^ ^]^ In addition to the above two types of materials, black phosphorus,^[^ ^136^ ^]^ MXene,^[^ ^137^ ^]^ and polydopamine (PDA)^[^ ^138^ ^]^ are often used as new light‐sensitive materials due to their high specific surface area and excellent biocompatibility. Due to the properties of semiconductor materials, it is easy to achieve the separation of electrons and holes under light conditions.^[^ ^139^ ^]^ The application of a single semiconductor is limited by the problems of fast recombination rates of electrons and holes, low light conversion ability, and low photocatalytic efficiency. To solve these problems, researchers further formed specific heterojunction structures between semiconductors, which reduced the probability of electron and hole recombination and greatly enhanced the photocatalytic ability.^[^ ^140^ ^]^ The presence of heterojunction structures enhanced the synergistic ability of photo heat and photodynamics and protected normal cells to a certain extent.

#### 4.2.2 Light responsive biomaterials controlled drug release

The drug‐controlled release is the most widely used photoresponsive biomaterial. According to the release principle, it is mainly divided into six categories: photothermal, photooxidation, photochemistry, photodissociation, photoisomerism, and photopolymerization.^[^ ^141^ ^]^ As ultraviolet light has certain harm to normal biological tissue, near‐infrared light is more widely used in the biomedical field. Therefore, we mainly review the photothermal and photooxidation systems using near‐infrared light as controlled release light.

The use of near‐infrared light to stimulate the response of biological materials is often accompanied by the generation of a photothermal effect,^[^ ^142^ ^]^ so this is a good control scheme for temperature sensitive materials. The common method of controlled release of biological materials in response to light stimulation is based on the relatively common normal physiological temperature of the human body. Thermally sensitive gel materials usually exhibit a low critical solution temperature near the physiological temperature, which ensures gel behavior at normal human temperature. In addition, the hydrogel state of the photothermal reaction can be rapidly transformed into a non‐flowing hydrogel state, resulting in controlled release so that the treatment can meet the actual needs.

Poly(*N*‐isopropyl acrylamide) (PNIPAM) monomer is commonly used to prepare thermoresponsive hydrogels because its critical solution temperature is about 32°C, which is close to the physiological temperature.^[^ ^143^ ^]^ Thermoresponsive hydrogels based on PNIPAM have also been widely reported. Mi et al. confirmed that when PNIPAM was copolymerized with other monomers, the addition of hydrophilic monomers increased the critical solution temperature, while the hydrophobic monomer decreased the critical point.^[^ ^144^ ^]^ Instead, it has been reported that by reducing temperature sensitivity, the stability of the hydrogel can be enhanced at low temperatures. In addition, the hydrophobicity of PNIPAM hydrogel increased with the increase in temperature, resulting in a decrease in swelling rate.^[^ ^145^ ^]^ Therefore, a thermosensitive drug‐release hydrogel dressing was prepared. In short, the hydrogel undergoes hydrophobic contraction at physiological temperatures above the critical solution temperature of PNIPAM, which then squelches out excess water and accelerates drug release, but no drug release at room temperature.^[^ ^146^ ^]^

**FIGURE 8:**
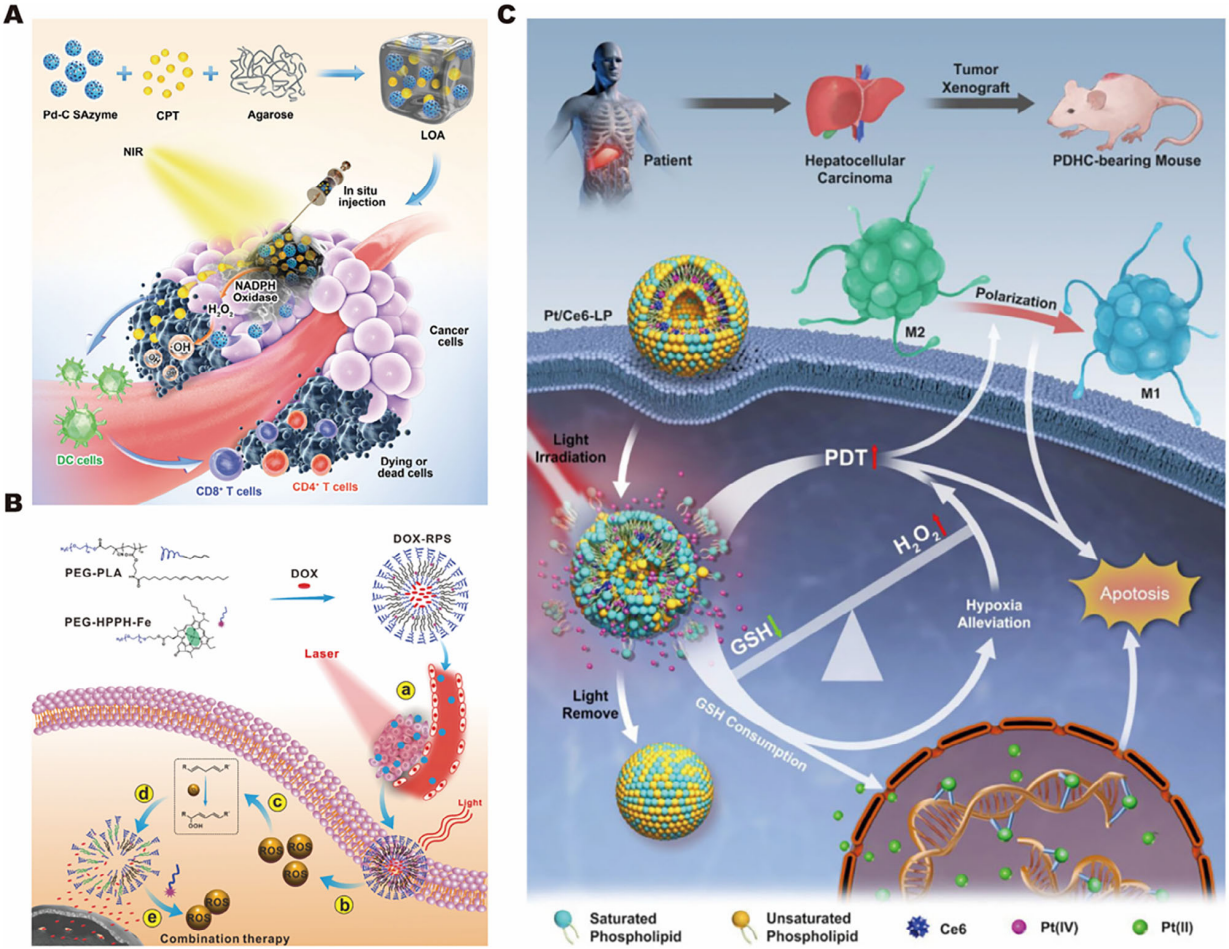
Photostimulation‐responsive biomaterial drug controlled release. (A) Low melting point gel intelligent photo controlled release system. Reproduced with permission.^[^ ^145^ ^]^ Copyright 2022, John Wiley & Sons. (B) Photocatalytic release system for reactive oxygen species regeneration. Reproduced with permission.^[^ ^149^ ^]^ Copyright 2021, John Wiley & Sons. (C) Photocatalytic release systems in anoxic environments. Reproduced with permission.^[^ ^150^ ^]^ Copyright 2021, Elsevier.

In addition to thermal response gels, it is also common to use materials with low melting‐points, such as agarose, as controlled release systems. Intelligent light‐controlled release systems have been prepared using low melting‐point agarose and pegylated BPNS. Under the condition of light, the nanostructure wrapped in the functional position changes from the gel state to the sol state to release the load.^[^ ^142^ ^]^ Controlled temperature conditions can not only control the rate of drug release but also control the effect on the cell membrane structure to a certain extent, which can be used to treat tumors or bacterial infections. As shown in Figure 8A, Pd‐C SAzyme and camptothecin (CPT) are co‐encapsulated in agarose hydrogel. When the hydrogel is stimulated with near‐infrared light, SAzyme can convert light into heat in response to the light stimulation, resulting in local hyperthermia and reversible hydrogel hydrolysis, releasing chemotherapy drugs.^[^ ^147^ ^]^

The controlled release system of photooxidation can produce singlet oxygen and hydroxyl free radicals in the process, and this singlet oxygen (unpaired electron pair) is highly reactive to most cells or molecular systems,^[^ ^148^ ^]^ so it has the advantage of synergistic treatment in tumor or bacterial infection sites. However, one limitation of reactive oxygen species response vectors is the need for large amounts of reactive oxygen species to be consumed during the triggering of drug release. However, this problem can be solved by photocatalysis of photoresponsive materials to produce large amounts of reactive oxygen species.^[^ ^149^ ^]^ However, due to the lack of oxygen in tumor sites, the reaction conditions for photocatalysis into reactive oxygen species are lacking, and the controlled release system subject to reactive oxygen species response will fail.^[^ ^150^ ^]^ Therefore, a photodynamics‐chemical kinetics cascade strategy for drug delivery nanosystem design is proposed. Coating the material in a self‐assembled photoresponsive polymer of poly(ethylene glycol)‐poly(lactic acid) (PEG‐PLA) and poly(ethylene glycol)‐(2‐(1‐hexyloxyethyl)−2‐devinyl pyropheophorbide‐α)‐iron chelate (PEG‐HPPH‐Fe) in response to light stimulation via the photosensitization agent HPPH will effectively generate reactive oxygen species, which further causes in situ oxidation of the linoleic acid chain, resulting in peroxidation of linoleic acid and changing the structural stability of the materials, allowing the triggering of drug release. Then, catalyzed by HPPH‐Fe, reactive oxygen species will regenerate from linoleic acid peroxide (LAP) through a Fenton‐like reaction, as shown in Figure 8B. Therefore, this photodynamic‐chemical kinetic cascade strategy does not require a large consumption of reactive oxygen species.^[^ ^151^ ^]^

Photoactivated liposomes are used as reactive oxygen species response drug‐controlled release vectors because unsaturated phospholipids in them can be reactive oxygen species, which damage the integrity of the liposomes. The researchers integrated the photosensitizer Ce6 and the chemotherapeutic drug Pt (IV) into the photoactivated liposome (Pt/Ce6‐LP), based on a Pt (IV) system where H_2_O_2_ would decompose and produce oxygen during the reduction of Pt (IV) to Pt (II). The improvement of photodynamic therapy efficiency and cisplatin resistance can also reduce the immunosuppression of the pro‐inflammatory M1‐tumor‐associated macrophages phenotype through polarization, as shown in Figure 8C.^[^ ^152^ ^]^ In addition, the incorporation of polyvalent metals, such as Mn (IV) and Ce (IV), can also decompose H_2_O_2_ into H_2_O and O_2_^[^ ^153^, ^154^, ^155^ ^]^ to alleviate the dilemma of cancer hypoxia.

The development of photoresponsive biomaterials from the earliest single function to the present multi‐function. From the earliest unconscious desire for a simple one‐time solution strategy to the realization that wound healing is a complex process and the conditions around the wound are constantly changing over time, which means that more strategies are needed to implement wound management and monitoring.

### 4.3 Application of biomaterials in response to ultrasonic stimulation

Ideal ultrasound techniques and responsive biomaterials can be used to achieve different aspects of treatment, such as dose, space, and time, while ensuring accuracy and effectiveness.^[^ ^156^ ^]^ At the same time, among all diagnostic imaging technologies, ultrasonic imaging has unique advantages such as real‐time, low cost, high safety, and easy access to portable devices,^[^ ^157^ ^]^ which can realize visual detection of damaged areas while undergoing repair and treatment. Therefore, ultrasonic stimulation response therapy has a natural advantage in in vivo applications.^[^ ^158^ ^]^

#### 4.3.1 Ultrasonic responsive biomaterials for tissue repair

The functional biomaterials of the ultrasonic stimulation response in terms of therapy and repair have the following advantages: (1) high spatiotemporal targeting. (2) Deep penetration of tissues and organs. (3) Image‐guided therapy. (4) High biosafety. (5) The miniaturization and portability of ultrasound devices have been widely used in clinical medicine.^[^ ^159^ ^]^ The ultrasonic stimulation usually used for treatment is low‐intensity pulsed ultrasound. When the ultrasonic mechanical wave propagates in the body medium, the piezoelectric material will generate a stimulating current, and the size and specifications of the responsive material implanted in the human body will be smaller than those of conventional electrical stimulation.^[^ ^160^ ^]^ Wu et al. prepared an ultrasonic generator, that mainly used potassium sodium niobate nanowires, PLLA, and poly(3‐hydroxybutyrate‐co‐3‐hydroxyvalerate), three common degradable piezoelectric materials, as degradable piezoelectric layer and packaged the generator with PLA and PCL. Finally, the degradable Mg electrode and Mo wire were used to complete the preparation of the ultrasonic response generator. The results show that the controlled generator with ultrasonic response can effectively promote nerve regeneration, and the whole system will not degrade too quickly in the process of nerve repair, resulting in implantation failure. However, the treatment system that separates the generator and nerve conduit always brings more risks and uncertainties.^[^ ^161^ ^]^ Therefore, Chen et al. implanted the neural guide catheter and ultrasonic response generator, which not only saved the implantation space in the body but also avoided the power loss caused by long distances.^[^ ^162^ ^]^ Other common piezoelectric materials such as PVDF,^[^ ^163^ ^]^ ZnO,^[^ ^164^ ^]^ BaTiO_3_,^[^ ^165^ ^]^ and other materials are also commonly used in ultrasonic response materials, but due to their poor biodegradation ability, they need to be removed from the patient's body after a second operation, which is often criticized by researchers. In addition, low‐intensity pulsed ultrasound can also affect the directed differentiation of bone marrow mesenchymal stem cells by enhancing the stromal cell‐derived factor‐1/C‐X‐C chemokine receptor type 4 (SDF‐1/CXCR4) signaling pathway and is also widely used in bone repair.^[^ ^166^ ^]^

The principle of sonodynamic therapy is similar to that of photodynamic therapy, but sonodynamic therapy has a better irradiation range. Zhang et al. used ultrasonic stimulation in response to PtCu nanoparticles to produce reactive oxygen species to kill bacteria around injured tissues and accelerate tissue repair.^[^ ^167^ ^]^ Similarly, sonodynamic therapy also has an obvious killing effect on cancer cells. Its advantage is that ultrasound therapy in the form of probes can accurately kill tumor tissue, but has little damage to neighboring healthy tissue.^[^ ^168^ ^]^

#### 4.3.2 Ultrasonic responsive biomaterials for controlled release

An accurate, controlled drug release system is conducive to a more rational application of drugs. According to the characteristics of ultrasonic stimulation, the controlled release system under ultrasonic stimulation is divided into four categories, as shown in Table 1.

(1) Micro/Nanobubbles

**TABLE 1:** Ultrasonic reaction system for controlling the release of substance.

Materials	Drug	Frequency	Application	Effectiveness	Reference
Micro/Nanobubbles	Phytoalexin/Chemotherapy drug	650 KHz–7 MHz	Bone repair/Tumor therapy	Reducing the expression of inflammatory factors (IL‐1β, TNF‐α, IL‐6)/ Tumor size was also reduced by about 60 percent	[171, 174]
Liposomes	Anticancer drug/Cell	237 KHz–1.3 MHz	Tumor therapy/Stroke treatment	The inhibitory activity of cancer cells was 29.16%/ Accelerated M2‐type polarization of microglia/ Total survivability increased by 2.5 times	[57, 180, 199, 200, 201]
Micelles	anticancer drug	500 KHz–1 MHz	Tumor therapy	The inhibitory activity of cancer cells was 36.5%/ The tumor inhibition ratio reached 93.84%	[190, 192]
Inorganic nanoparticles	Anticancer drug	0.5 MHz –1.6 MHz	Tumor therapy	Apoptosis is obvious	[197, 198, 202]

Microbubbles have been widely used as contrast agents for ultrasonic imaging.^[^ ^169^ ^]^ More importantly, when stimulated by frequencies that approximate resonance, microbubbles oscillate like cavitation bubbles and may eventually burst.^[^ ^170^ ^]^ Therefore, it is often used as a controlled release system. Bai et al. wrapped phytoalexin in nano‐bubbles, regulated the release of phytoalexin in nano‐bubbles by ultrasound, and accurately suppressed excessive peak immune responses within 24–48 h after injury, thus interfering in bone tissue repair.^[^ ^171^ ^]^ In addition to conventional drug‐controlled release, micro/nanobubbles are often used for drug‐controlled release that needs to cross the blood‐brain barrier.^[^ ^172^ ^]^ The enhanced cavitation of the ultrasonic by injecting microbubbles also enables resonation‐induced ultrasound between the ultrasonic and microbubbles, increasing the permeability of the blood‐brain barrier and making it easier to pass the blood‐brain barrier.^[^ ^173^ ^]^ Xiao et al. used high‐intensity focused ultrasound oscillations to break the nanobubbles, which at the same time produced a transient impact on the blood‐brain barrier and allowed drugs to bypass it and reach the brain. FePt was transported to the brain together with doxorubicin (DOX) in the nanobubbles, which also enabled long‐term magnetic resonance imaging tracking and a high signal‐to‐noise ratio. Effective imaging and tracking of brain tumors.^[^ ^174^ ^]^

(2) Liposomes

Microbubbles are not suitable for tumor targeting and storage retention due to their large size.^[^ ^175^, ^176^ ^]^ Therefore, researchers have further studied and found nano‐liposomes, which have the advantages of small size, large specific surface area, good pharmacokinetics, excellent targeting ability, accessible surface functionalization, and so on, making them highly popular in controlled release.^[^ ^177^ ^]^

**FIGURE 9:**
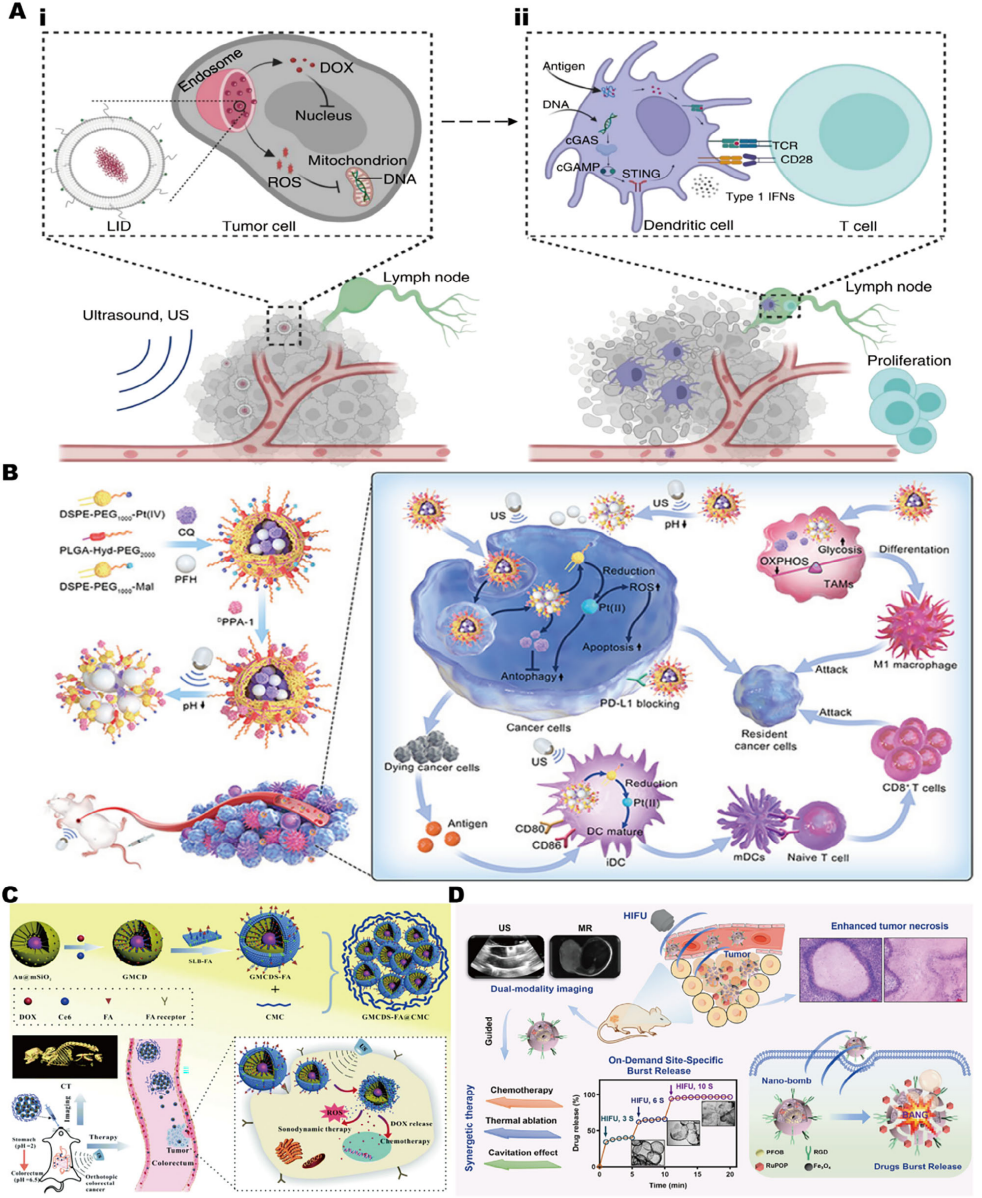
Ultrasonic stimulation‐response biomaterial for controlled release drugs. (A) liposomes loaded with indocyanine green and doxorubicin: (i) activation of cGAS‐STING signaling; (ii) induction of T cell immunity. Reproduced with permission.^[^ ^177^ ^]^ Copyright 2023, Spring Nature. (B) Dual‐response controlled release system regulates macrophage activation and T cell immunity. Reproduced with permission.^[^ ^190^ ^]^ Copyright 2022, American Chemical Society. (C) Controlled release system of CT imaging based on double stimulus response. Reproduced with permission.^[^ ^195^ ^]^ Copyright 2021, Royal Society of Chemistry. (D) Blasting release system for ultrasound/magnetic resonance dual‐mode imaging. Reproduced with permission.^[^ ^196^ ^]^ Copyright 2021, Elsevier.

Liposomes loaded with indocyanine green and DOX can enhance the delivery of DOX under ultrasonic stimulation, and the reactive oxygen species produced can induce the oxidation of tumor mitochondrial deoxyribonucleic acid (DNA) and promote the transfer of oxidized tumor mitochondrial DNA to antigen‐presenting cells (APCs), to effectively activate cGAS‐STING signal transduction.^[^ ^178^ ^]^ In addition, liposomes induce effective anti‐tumor T cell immunity in response to ultrasonic stimulation, and tumor regression,^[^ ^179^ ^]^ is shown in Figure 9A. At the same time, hybrid liposome nanocarriers based on polyvinylidene‐trifluoro ethylene, graphene carbon quantum dots, and a hydrophobic drug can combine controlled release drug delivery with fluorescence imaging of cancer cells to achieve imaging therapy and significantly enhance the effect of drug use.^[^ ^180^ ^]^

Although the volume of liposomes is significantly smaller than that of microbubbles, liposomes still cannot directly cross the blood‐brain barrier into the brain, and cavitation is still required to be induced by long‐pulse ultrasound (pulse length: > 1 ms) and microbubbles, resulting in the temporary opening of the tight connection of the blood‐brain barrier to achieve liposomes delivery,^[^ ^181^ ^]^ which is of great significance in the treatment of brain diseases. Kim et al. used focused ultrasound and microvesicles to successfully transport DOX‐loaded liposomes into the brain across the blood‐brain barrier to achieve efficient treatment of glioblastoma.^[^ ^182^ ^]^

In addition to conventional liposomes, thermosensitive liposomes are considered the second generation of liposomal carriers. These liposomes are activated by heat and release most of their contents in a temperature range of 40–43°C.^[^ ^183^ ^]^ Due to the destruction of the microvascular network in the focal area, effective drug delivery is hindered, and chemotherapy‐induced cell death is reduced.^[^ ^184^ ^]^ Thermosensitive liposomes containing the anti‐cancer drug doxorubicin combined with high‐intensity focused ultrasound will deliver targeted drugs to overcome the shortcomings of conventional chemotherapy. The results showed that thermosensitive liposome‐mediated drug delivery led to a more than 100% increase in cell death by providing high cycle times and improved bioavailability. In addition, increasing sound power can enhance the desired effect, namely heat necrosis in the central area of the tumor, greatly improving the efficacy of anti‐cancer drugs.^[^ ^183^ ^]^

Image‐guided thermosensitive liposomes can achieve higher drug therapy efficiency. Thanou's team previously investigated dual‐labeled magnetic resonance imaging and near‐infrared fluorescence tracking of heat‐sensitive liposomal loading systems that reach focal areas, enabling a rapid response to focus‐induced.^[^ ^185^ ^]^ As the research progressed, the team developed a more advanced visualization dual‐drug loading system. The clearance and distribution of the drug loading system were controlled in mice after near‐infrared fluorescence and magnetic resonance imaging monitoring, and the survival time of the mouse model was greatly extended to 2.5 times.^[^ ^57^ ^]^

Although the targeting and controlled drug release ability of liposomes can improve the drug use effect, the current poor acoustic response ability of liposome carriers leads to low drug release efficiency, resulting in a certain degree of resource waste. The release efficiency of carbon dioxide‐carrying liposomes synthesized by supercritical carbon dioxide (CO_2_) is 17.1 times higher than that of liposomes synthesized by the conventional Bangham method. Meanwhile, the release efficiency of CO_2_‐bearing liposomes synthesized by supercritical CO_2_ and monoethanolamine is 19.8 times higher than that synthesized by the Bangham method. These studies on the efficiency of acoustic liposome release provide an alternative liposome synthesis strategy for on‐demand drug release by ultrasound irradiation in future therapies.^[^ ^186^ ^]^

(3) Micelles

Supramolecular micelles are composed of non‐covalent bonds. Because of the properties of non‐covalent bonds, the function of micelles is more flexible and it have a wider range of application scenarios.^[^ ^187^, ^188^ ^]^ Single‐molecule micelles are of interest as controlled‐release systems because of their higher stability compared to supramolecular micelles.^[^ ^189^ ^]^ The spirochaete micelles are made of a light‐solidifiable and biodegradable polyethylene glycol diacrylate‐pentaerythritol triacrylate with porous micellar surfaces that increase surface area and facilitate drug release by a focused ultrasonic beam, which increases its release by 2.5 times.^[^ ^190^ ^]^ Free drugs in the blood are easily removed or broken down, and PLGA nanoparticles have been shown to have the sustained release effect of encapsulated drugs.^[^ ^191^ ^]^ To ensure the stability of the nanoparticles' properties in the blood circulation, the pH and glutathione double‐response nanoparticles were modified with PEG. Once exposed to a low‐acidity tumor microenvironment, nanoparticles may break hydrazone bonds and be exposed,^[^ ^192^ ^]^ as shown in Figure 9B. To precisely target drug delivery, a new type of dendritic polyurethane prodrug with a drug content of 18.9% was prepared by coupling DOX to the end group of functionalized dendritic polyurethane through acid‐unstable imine bonds, which can easily form a single‐molecule micelle. Compared with non‐covalently loaded single‐molecule micelles, it has stronger drug binding energy, lower drug loss during transportation, and exhibits excellent pH/ultrasonic dual trigger drug release performance, with ultrasonic response attributed to its unique strawberry topology. This characteristic gives them broad potential for precise local drug delivery.^[^ ^193^ ^]^

(4) Inorganic nanoparticles

Liposomes, organic microvesicles, or micelles have been used to improve the efficacy of focused ultrasound therapy.^[^ ^194^, ^195^ ^]^ However, the weak rigidity and low thermal resistance of organic carriers can easily lead to drug leakage, and the large particle size also becomes a limitation of its clinical application. Therefore, it is necessary to develop inorganic nanoparticles as ultrasonic responsive load systems.^[^ ^196^ ^]^

For patients with inflammatory colorectal cancer, drug therapy is extremely difficult due to the deep treatment location and the restriction of the gastric acid environment. Meanwhile, due to the unique structural structure of the gastrointestinal tract, the focal targeting of an organic drug‐loading system with weak stiffness is also very difficult. Therefore, the use of mesoporous silicon coated with gold nanoparticles and loaded with chlorine and DOX, combined with folic acid‐modified phospholipid, can achieve pH/ultrasonic dual response, step targeting, and precisely controlled release of enteric‐soluble particles. In addition, gold nanoparticles in mesoporous silicon can be used for CT imaging, which can guide treatment. It provides a new approach for the treatment and diagnosis of in situ colorectal cancer,^[^ ^197^ ^]^ such as shown in Figure 9C. By coating the PLGA surface with perfluorinated carbon hydrophobic anti‐tumor ruthenium complex and superparamagnetic Fe_3_O_4_, ultrasound/magnetic resonance imaging dual‐mode imaging was achieved. At the same time, perfluorinated carbon caused the collapse of the ultra‐thin silicon shell in response to the super stimulus. Thus, the strong mechanical stress during blasting was enhanced the therapeutic effect was enhanced, and residual tumors induced by uneven high‐intensity focused ultrasound ablation were suppressed to the maximum extent, as shown in Figure 9D.^[^ ^198^ ^]^

### 4.4 Application of biomaterials in response to magnetic stimulation

#### 4.4.1 Magnetic stimulation response biomaterials for tissue repair

Tissue repair applied by magnetic stimulation biomaterials mainly includes simple transfer behavior,^[^ ^203^ ^]^ magnetoelectricity,^[^ ^11^, ^204^ ^]^ and magnetothermal^[^ ^205^, ^206^ ^]^ as shown in Table 2. Studies have shown that In the recovery of muscle function health, the use of magnetic drive to stimulate the orderly arrangement of muscle cells showed a positive therapeutic effect^[^ ^207^, ^208^ ^]^ and the application of an appropriate magnetic field at the site of nerve trauma can also have a highly positive effect on its regenerative ability. After implanting magnetic‐responsive scaffolds covered with magnets, the use of small magnetic stimulation in vitro can achieve a good therapeutic effect.^[^ ^209^, ^210^ ^]^ In addition, Wang et al. made use of the endocytosis make Fe_3_O_4_ into Schwann cells, thus enabling Schwann cells to have the ability of magnetic migration, and carrying out orderly migration of Schwann cells by using external magnetic fields, greatly promoting axon regeneration.^[^ ^211^ ^]^

**FIGURE 10:**
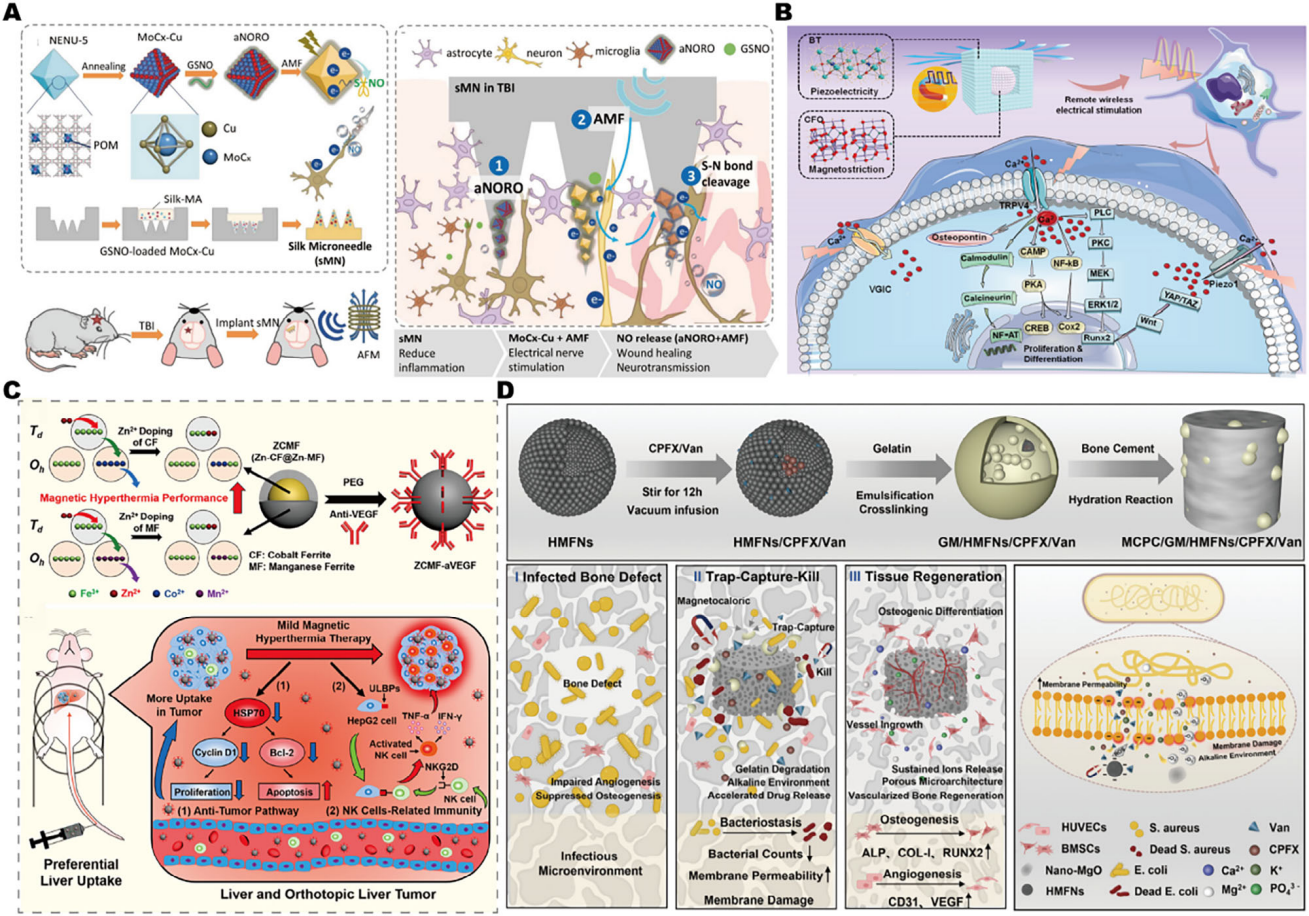
Application of magnetic stimulation‐response biomaterials in tissue repair. (A) Collaborative regeneration of chemical and electrical signals based on magnetic response. Reproduced with permission.^[^ ^209^ ^]^ Copyright 2023, Elsevier. (B) Magnetic response electrical signals promote endogenous response and promote bone regeneration. Reproduced with permission.^[^ ^216^ ^]^ Copyright 2020, Elsevier. C. Magnetic response heat signal promotes endogenous response to kill cancer cells. Reproduced with permission.^[^ ^218^ ^]^ Copyright 2021, American Chemical Society. (D) Aggregation‐capture‐kill system based on magnetic response. Reproduced with permission.^[^ ^223^ ^]^ Copyright 2023, John Wiley & Sons.

1. Magnetoelectric effect: Compared with ordinary electric stimulation, it can achieve a simpler and more convenient treatment.^[^ ^212^ ^]^ S‐nitrosoglutathione and MoCx‐Cu are used to form a magnetic response octahedron, which will trigger the release of nitric oxide (NO) and produce magnetoelectric effects. Such implantable devices for acute brain trauma repair provide NO and electron mobility on demand to promote angiogenesis, nerve regeneration, and functional recovery in vivo, as shown in Figure 10A.^[^ ^213^ ^]^ However, conventional magnetoelectricity is believed to give limited electrical stimulation ability, so piezoelectric materials are introduced to seek more significant electrical stimulation ability.^[^ ^214^ ^]^ It is worth noting that the injured site of the patient is fixed and cannot move or move during the actual clinical repair process, which means that there is not enough stress to activate the piezoelectric material to generate electrical signals.^[^ ^215^ ^]^ Therefore, a magnetostrictive material that can move and rotate between magnetic domains in response to an external magnetic field, thus stretching and deforming the material in length and volume, has been widely concerned.^[^ ^216^ ^]^ The core‐shell nanoparticles were formed by the in situ growth of piezoelectric BaTiO_3_ on magnetostrictive CoFe_2_O_4_. Among them, the CoFe_2_O_4_ magnetic core can generate strain in response to the magnetic field, and the resulting strain is transferred to the BaTiO_3_ shell through interface coupling, forcing the BaTiO_3_ domain to reverse, and finally inducing the generation of electrical signals. Encouragingly, when stimulated by a magnetic field, the material was able to generate a voltage of 100 mV cm^−1^, putting it within the appropriate range of electrical stimulation (50–150 mV cm^−1^). At the same time, significant electrical signals change the Ca^2+^ concentration and membrane charge balance inside and outside the cell, The membrane tension changes activate the mechanically sensitive ion channel component 1,^[^ ^217^ ^]^ transient receptor potential vanilloid 4 ion channel^[^ ^218^ ^]^ and the voltage‐gated ion channel. The Ca^2+^ influx further regulates the protein kinase signaling pathway, accelerates the release of osteopontin, and promotes cell differentiation. At the same time, Piezo1 can also accelerate cell differentiation by regulating the co‐activation of runt‐related transcription factor 2 (RUNX2)‐related proteins and transcription processes.^[^ ^219^ ^]^ In addition, the activation‐dependent dephosphorylation of activated nuclear factor NF‐AT, which affects transient receptor potential vanilloid 4‐mediated Ca^2+^ calcineurin, promotes cell proliferation and differentiation, indicating the possibility of good bone repair, as shown in Figure 10B.^[^ ^220^ ^]^
2. Magnetothermal effect: Magnetothermal is not limited by penetration depth, nor is it attenuated by tissue, and the ability to fine‐control local temperature with nanoscale spatial resolution in a remotely controlled manner is a promising application in biomedical engineering.^[^ ^221^ ^]^

**TABLE 2:** Types and applications of biomaterials with magnetic stimulation response.

Material	Type	Application	Mechanism	Reference
CoFe_2_O_4_@BaTiO_3_/ [P(VDF‐TrFE)]	Magnetoelectricity	Bone repair	Promote the differentiation of BMSC	[230, 231]
PLLA/CFO@BT	Magnetoelectricity	Bone repair	Stimulate cell function expression, improve proliferation and differentiation	[220]
CoFe_2_O_4_/PVDF	Magnetoelectricity	Bone repair	Promote osteoblast proliferation	[232]
MoCx‐Cu	Magnetoelectricity	Brain injury repair	Stimulate NSC differentiation and growth	[213]
CFO@BFO	Magnetoelectricity	Nerve repair	Transfer neuron cells, stimulate differentiation	[233]
Fe_3_O_4_	Magnetoelectricity/Magnetothermal	Nerve repair/Cancer treatment/Bacteria kill	Promote Schwann cell migration/Alkyl free radical release/Reactive oxygen species	[211, 227, 234]
CoFe_2_O_4_@Zn‐MnFe_2_O_4_	Magnetothermal	Cancer treatment	Related protein regulation	[222, 235]
Bi_35_In_48.6_Sn_16_Zn_0.4_	Magnetothermal	Relieve pain	Regulates immune factors and membrane protein receptors	[236]
Magnetic Bioglass	Magnetothermal	Cancer treatment	Death from Ca^2+^ overload	[226]
Fe_3_O_4_+GO	Magnetothermal	Cancer treatment	Temperature kills tumors	[237]
Fe_3_O_4_@DPA/HA	Magnetothermal	Cancer treatment	Promote immune activation	[238]

Researchers have synthesized a core‐shell structure of Zn^2+^ doped Zn‐CoFe_2_O_4_@Zn‐MnFe_2_O_4_ nanoparticles.^[^ ^222^ ^]^ Due to the exchange coupling magnetic properties between the core‐shell and the doping of Zn^2+^, it has excellent controllable magnetic response hyperthermia performance and will inhibit the expression of heat shock protein 70,^[^ ^223^ ^]^ while enabling liver cancer cells to activate natural killer cells to clear cancer cells by significantly upregulating the expression of UL16‐binding protein^[^ ^224^ ^]^ as shown in Figure 10C. However, in the routine process of tumor repair, the invasion of normal cells by tumor cells leads to the occupation of normal tissues by cancer cells. Therefore, in the process of killing cancer cells, the defect of normal tissues will inevitably be caused. Therefore, the treatment of tumors requires not only the removal of cancer cells but also the repair of normal tissues.^[^ ^225^ ^]^ A composite scaffold (CS‐MBG) was formed by combining the high‐porosity magnetic biology with calcium sulfate (CS) bone cement. Under magnetic field conditions in vitro, CS‐MBG will release Ca^2+^ leading to cell death and calcium overload. At the same time, the osteogenic activity of CS‐MBG can promote postoperative bone repair to kill two birds with one stone.^[^ ^226^ ^]^ Dai et al. constructed drug‐carrying hollow mesoporous ferrite nanoparticles with pomegranate structure and prepared them into calcium magnesium phosphate cement. Interestingly, the positively charged gelatin can act as a trap, and MgO can effectively kill bacteria under the action of an alternately magnetic field and multi‐directional synergistic action. The collection‐capture‐kill system was constructed as shown in Figure 10D.^[^ ^227^ ^]^

**FIGURE 11:**
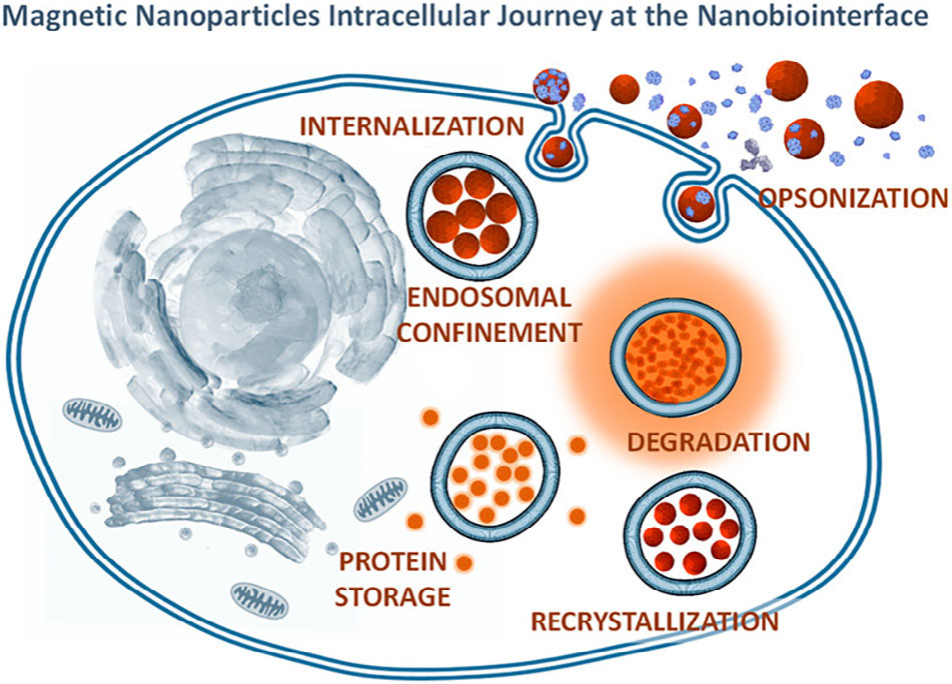
Potential biosafety risks of nanoparticles: Acid degradation and recrystallization. Reproduced with permission.^[^ ^204^ ^]^ Copyright 2020, American Chemical Society.

According to the characteristics of magnetically responsive biomaterials, some researchers have proposed visualization of the therapeutic process.^[^ ^228^ ^]^ This dual synergistic function of treatment and imaging will be the focus of future repair research. However, the toxicity of magnetic nanoparticles is the primary problem that needs to be solved. It is generally believed that the toxicity of nanomaterials is determined by many factors, such as size, surface area, surface modification, and aggregation state.^[^ ^229^ ^]^ As the magnetically responsive biomaterials mostly function in vivo through intravenous injection, most of the magnetic nanoparticles accumulate in the liver and spleen. In addition, magnetic nanoparticles are usually mediated by lysosomes and release ions. The release of ions promotes the enhancement of the intracellular oxidation reaction, and its balance is highly regulated by the internal enzyme system and recrystallization is also associated, so their biosafety needs further exploration, as shown in Figure 11.^[^ ^206^ ^]^

#### 4.4.2 Magnetic stimulation response biomaterials for targeting and controlled release

The ideal multifunctional nanocomposite response system can provide clear biomedical applications in repeated space and time and the ability to remotely control and regulate cell behavior with high precision.^[^ ^239^ ^]^ In this regard, magnetic nanomaterials have attracted much attention due to their unique magnetic response applications, for example, the transmission and release of deformation over time.^[^ ^240^ ^]^ Most materials are used in penetration fields, including the human body, and can achieve precise spatial responses.^[^ ^241^ ^]^

**FIGURE 12:**
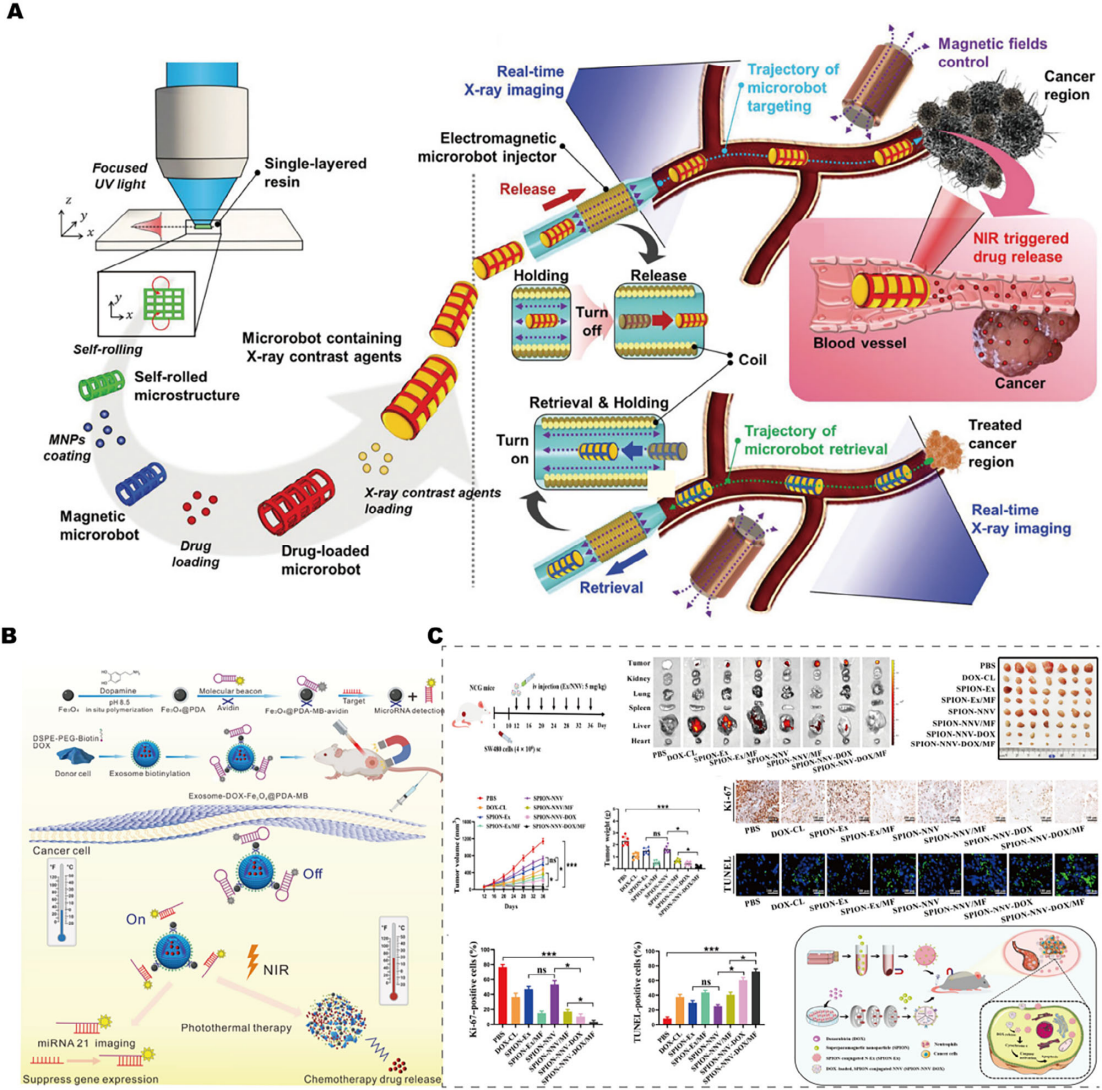
Magnetic stimulation response biomaterial targeting and drug controlled release. (A) Recoverable magnetic response controlled release system. Reproduced with permission.^[^ ^241^ ^]^ Copyright 2021, John Wiley & Sons. (B) Fluorescence imaging based on magnetic stimulation response and triple synergistic tumor therapy. Reproduced with permission.^[^ ^244^ ^]^ Copyright 2020, Elsevier. (C) Magnetic stimulation in response to endogenous responses to tumor therapy. Reproduced with permission.^[^ ^246^ ^]^ Copyright 2022, American Association for the Advancement of Science.

Construction of a simple magnetic response controlled release system. For example, Giron et al. constructed a magnetic response gel containing nerve growth factor inside the neural guide catheter to control the distribution and degradation of growth factor inside the gel by controlling the external magnetic field.^[^ ^242^ ^]^ In addition to magnetically responsive controlled release, the application of its targeting ability is also essential. Huang et al. loaded growth factors into magnetically responsive PLGA and used gels with sol‐gel conversion ability to achieve gradient release and target drive under the control of magnetic field.^[^ ^243^ ^]^ Due to its good paramagnetization, Fe_3_O_4_ can be targeted to move to any position under the action of the external magnetic field and can physically destroy biofilm during movement. The movement of a large number of Fe_3_O_4_ nanoparticles can enhance its effect and reduce bacterial adhesion. The •OH produced by Fe_3_O_4_, which also has a higher specific surface area, is more conducive to penetration into deeper layers of biofilms.^[^ ^244^ ^]^ To solve the problem of cytotoxicity of magnetic nanoparticles mentioned above, researchers have reduced concerns about the toxicity of biodegradable materials through the recycling of materials,^[^ ^245^ ^]^ as shown in Figure 12A.

Furthermore, creating a treatment platform by combining various materials and organisms can help minimize harmful side effects. Exosomes, a commonly occurring biomass, are particularly effective in treating tumors due to their excellent drug‐carrying capabilities, natural tumor‐targeting abilities, and capacity to induce endogenous reactions.^[^ ^246^ ^]^ Fe_3_O_4_@PDA allows exosomes to target accumulation at tumor sites under magnetic field action. Fe_3_O_4_@PDA can strongly quench the fluorescence of molecular beacons, and at the same time, specifically recognize microRNA‐21, an intracellular tumor diagnostic and prognostic marker.^[^ ^247^ ^]^ The fluorescence of molecular beacons is turned on again, and fluorescence imaging and molecular, chemical, and chemical functions of microRNA‐21 are performed simultaneously by forming a double‐stranded structure and inhibiting the function of microRNA‐21. Temperature triple synergistic antitumor therapy^[^ ^248^ ^]^ is shown in Figure 12B. Exosomes of neutrophils can induce tumor cell apoptosis by delivering cytotoxic proteins and activating caspase signaling pathways.^[^ ^249^ ^]^ It can be modified by superparamagnetic iron oxide nanoparticles to achieve a higher tumor‐targeted therapy effect and achieve effective tumor inhibition^[^ ^250^ ^]^ as shown in Figure 12C. This engineered design provides a powerful, efficient, and safe nano platform for drug delivery for cancer treatment.

## 5 SUMMARY AND OUTLOOK

In this review, we focus on the unique ability of externally applied stimuli to influence the manipulation of engineered biomaterials. Various types of stimulation, including electrical, light, ultrasonic, and magnetic, each have their advantages and can be used as controlled triggers in different ways.

(1) Advantages and disadvantages of different physical stimulation methods: Depending on the stimulating energy, the development of therapeutic uses in vivo can take different forms, each with its advantages and limitations for use in vivo. Electrical stimulation, for example, can be used as a preconditioning method before clinical implantation, as it has been shown to influence long‐term differentiation with short periods of stimulation. Although electric fields can be easily applied to external settings, direct electrical stimulation requires more invasive methods to connect to an electrically responsive scaffold. Specific stimulation sites, such as conductive materials, are typically used in bone and muscle environments since these tissues can receive natural electrical stimulation and tend to show better therapeutic results. Light‐responsive systems can deliver significant results at shallower depths but have more limited signal penetration and focusing capabilities for transdermal applications, which limits their use at non‐shallow tissue depths. Nevertheless, light‐activated platforms still have clinical potential in vivo, especially for surface sites, where light‐responsive systems can more finely control intracellular reactions in terms of biochemical reactions. Light‐responsive systems also show high utility in regulating the mechanical properties of materials by regulating chemical crosslinking or photo‐mediated degradation. In contrast, ultrasonic and magnetic systems benefit from excellent tissue permeability and are therefore ideally suited to drive materials remotely after implantation to stimulate bioactive agent delivery or other therapeutic effects in vivo with more frequent targeting, diagnostic, and controlled release applications involving stimulated emission from post‐incorporation. Compared to ultrasonic systems, magnetically responsive biomaterials are less capable of delivering drugs with spatial precision because the stimulating energy is conducted throughout the biomaterial or generated by external fields.
(2) The suitability and safety of responsive biomaterials: In the field of responsive biomaterials, it is of utmost importance to carefully deliberate the efficacy and appropriateness of materials for the specific site of usage. In addition, biosafety and patient ethics are key factors to consider in this field. The incorporation of metal ions and their compounds in various stimuli‐responsive biomaterials requires meticulous experimentation to assess whether degradation within the body could potentially produce harmful heavy metal ions. Moreover, researchers must exercise caution regarding the possibility of nanotoxicity, as these materials are often micro‐ or nano‐sized. It is crucial to take all these factors into account to ensure the utmost safety and effectiveness of the biomaterials in use.
(3) Clinical transformation: The medical and health platforms are continuously evolving, and research and development of biological materials that respond to physical stimulation are underway. This field shows promising results in tissue repair, drug delivery, and diagnostics. Various manufacturing methods such as 3D printing, microfluidics, electrospinning, and microencapsulation are employed to prepare biomaterials that can sense and respond to physical stimuli. However, the challenge is to move these materials from the lab to clinical applications, as successful laboratory results in vivo have not yet been translated into the clinic. Despite great progress in this area, much effort is still required to develop practical applications for these materials.
(4) Development goals and directions: The external stimulus‐response platform has a solid potential to drive biomedical advances, and with the combination of various stimuli, more responsive materials can be built. Exciting possibilities exist for synergistically combining multiple stimuli to precisely control multiple environmental features. Establishing new personalized medical technology can provide new goals and directions for future medical technology development.

## CONFLICT OF INTEREST STATEMENT

The authors declare no conflicts of interest. Xiaoyuan Ji is a member of the *Exploration* editorial board.
